# Supplementary material for: Comparison of World Health Organization and Demographic and Health Surveys data to estimate sub-national deworming coverage in pre-school aged children
Source: PLoS Negl Trop Dis. 2020 Aug 17;14(8):e0008551. doi: 10.1371/journal.pntd.0008551 (PMC7462292; doi:10.1371/journal.pntd.0008551)
Supplement: S1 Technical Appendix — (DOCX) [file pntd.0008551.s002.docx]

- **S1 Technical appendix**

In this supplement, we provide additional methodologic details for estimating deworming coverage from data reported to the World Health Organization (WHO) and by using the Demographic and Health Surveys (DHS) data.

*Deworming estimates using data reported to WHO*

We utilized deworming campaign coverage data reported to WHO by each country’s Ministry of Health. We estimated coverage using WHO data on the proportion of total deworming medications for each treatment campaign and the number of pre-school aged children in each DHS survey. In districts with biannual deworming campaigns, where both campaigns met inclusion criteria, we estimated a single treatment probability (coverage) for pre-school aged children per district as a function of coverage estimates from both overlapping campaigns reported to WHO. Any deworming campaigns reported to WHO with no corresponding DHS interviews conducted within the recall period following campaign initiation were excluded from analysis. In summary, one deworming coverage was estimated from WHO data per district with included DHS and WHO data. Below we list the equations used to calculate deworming coverage per district.

- Equations 1-2 were used to calculate district-level deworming coverage ($\mu_{r})$ for districts with children eligible for either one or two overlapping deworming campaigns reported to WHO:
- $\left[ 1 \right] \mu_{r}=\left\{ \begin{aligned} \frac{N_{medications}}{N_{children}} | &N_{campaigns}=1 \\ 1- \rho\left( \alpha\right) | &N_{campaigns}=2 \end{aligned} \right.$
- $\left[ 2 \right] \rho\left( \alpha\right)={(1-P}_{1})*\left( 1-P_{2} \right)+\left( (\alpha)*\sqrt{P_{1}*\left( 1- P_{1} \right)*P_{2}*\left( 1-P_{2} \right)} \right)$

${where P}_{1}=\frac{N_{medications , 1}}{N_{children , 1}} ; P_{2}=\frac{N_{medications , 2}}{N_{children , 2}}$

- Deworming coverage estimates using WHO data were calculated using one of two methods, based on the number of eligible campaigns for a given district. For districts where all children included in DHS were eligible for only one deworming campaign during the recall period for each DHS interview, we estimated coverage with equation (1) and as outlined in the Methodology section. For districts where some children included in DHS were eligible for two campaigns overlapping during the recall period for some DHS interviews, we estimated the district-level deworming coverage using a linear combination of the reported coverage for each of the overlapping campaign based on an estimated correlation coefficient and assumption of a binomial distribution (equation 2). We bounded the district-level deworming coverage between the greater of either individual campaign ($N_{Campaign, 1})$ as the lower bound and the sum of coverage reported for both campaigns or 100% as the upper bound. We established a “treatment correlation” co-efficient, $\alpha$, to account for the correlation in coverage between both overlapping campaigns. The coefficient provided a measure for the correlation between the targeted population of both deworming campaigns which was used to estimate the probability a given child was treated twice $(\rho$). We assumed deworming coverage across campaigns was correlated to some unknown degree and varied the correlation in sensitivity analyses. $(1-\rho\left( \alpha\right))$ in equation (1) represents the total annual proportion of unique pre-school aged children per district who received deworming medication over two overlapping campaigns while accounting for repeat medication.

Equation 3 was used to fit the treatment correlation value, $\alpha$, by minimizing the sum of squares error (SSE) for all districts between deworming coverage estimates using data reported to WHO and by DHS.

- $\left[ 3 \right] SSE= \sum_{r=1}^{regions} {(\bar{x_{r}} - \mu_{r})}^{2}$

We fit the “treatment correlation” parameter, $\alpha$, by minimizing the sum of squares error across all differences in district-level deworming coverage reported to WHO and estimated by DHS. We fit the parameter over 250,000 trials until convergence (See Figure A1).

- *Deworming estimates using data provided by DHS*

We used individual-level survey data collected from maternal respondents, serving as proxies for their children, to collect information on relevant variables, including pre-school child deworming receipt status. We relied on responses to survey questions from the DHS-VI questionnaire (See Table A1) to determine district-level deworming coverage in pre-school aged children. Responses were filtered to include only children over 12 months in age at time of deworming based on the date deworming campaigns were initiated in their district of residence. We only included responses to deworming status collected within an established recall period following the initiation date for deworming campaigns reported to WHO. Below we list the equations used to estimate mean deworming coverage and standard deviation per district.

Equations 4 and 5 were used to estimate the district-level deworming coverage by DHS and its standard deviation, respectively:

- $\left[ 4 \right] \bar{x_{r}}= \frac{N_{Yes} + {(w_{maybe}*N}_{Maybe})}{N_{Yes} + N_{Maybe} + N_{No}}$
- $\left[ 5 \right] \bar{\sigma_{r}}= \sqrt{\frac{\bar{x_{r}}*(1-\bar{x_{r}})}{N_{Yes} + N_{Maybe} + N_{No}}}$
- The district-level deworming coverage estimates were calculated as the proportion of total “Yes” and “Maybe” responses (weighted by probability of deworming receipt, base case of +0.5) over all eligible responses for child deworming receipt confirmed within the recall period of an established deworming campaign’s start date. The standard deviation per district-level deworming coverage estimate was calculated assuming a binary distribution of responses. When calculating national-level deworming coverage and standard errors, DHS reported survey weights were incorporated.

All analyses were programmed in R version 3.5.0 (R Foundation for Statistical Computing; Vienna, Austria) and conducted in Stata/IC version 15.1 (StataCorp LP; College Station, United States), with the files and instructions for use available online (see main text reference to Github repository).

- *Additional descriptive results*
- In Burundi, the mean district-level DHS sample based the base 6 month recall period was 526 pre-school aged children (range: N=393 in Bujumbura Mairie to N=647 in Muyinga, see Figure 3) where a mean of 50.1% of children per district sampled were girls (range: 46.1% in Bujumbura Mairie to 54.2% in Mwaro). In Myanmar, the mean district-level DHS sample size in the base case was 133 pre-school aged children (range: N= 99 in Sagaing to N=181 in Kayin) where a mean of 48.1% of children sampled per district were girls (range: 40.5% in Ayeyarwaddy to 53.0% in Taninthayi) based on the sampling frame. In the Philippines, the mean district-level DHS sample size in the base case was 475 pre-school aged children (range: N= 252 in Ilocos to N=640 in Bangsamoro) where a mean of 47.4% of children sampled per district were girls (range: 44.1% in Cagayan Valley to 53.7% in Bicol). Based on a 6-month recall period, 58.8% of pre-school aged children in Burundi were eligible for exactly one deworming campaign with the remaining 41.2% eligible for two deworming campaigns. In Myanmar and the Philippines, all pre-school aged children were eligible for only one deworming campaign based on a 6-month recall period.
- *Lymphatic filariasis sub-analysis*

To analyze the effects of albendazole receipt for deworming delivered outside of large scale programmatic treatment, also known as mass drug administration (MDA) campaigns that were only captured by the maternally-reported deworming DHS data, we performed a sub-analysis assessing the mean absolute difference in district-level coverage by data source stratified by presence of programmatic treatment for lymphatic filariasis, a proxy for other public health programs. We first categorized all included districts per country endemic to lymphatic filariasis during the observed deworming timeframe by level of endemicity to lymphatic filariasis, using data provided by WHO on presence of sub-district-level (administrative level II) treatment campaigns. We stratify districts using three definitions to lymphatic filariasis endemicity. Districts were first stratified by whether 50% of sub-districts received lymphatic filariasis treatment. Second, districts were binned by percent of sub-districts receiving lymphatic filariasis treatment (0%, 1-50%, 51-99%, 100%). Finally, districts were stratified by whether any of their sub-districts received lymphatic filariasis treatment. As treatment for lymphatic filariasis is only recommended for children 2 years of age and older, we only include DHS deworming data for pre-school aged children of 2-4 years in age at time of deworming. For each stratification method, we calculated the mean absolute difference in district-level deworming coverage between data sources and the respective standard deviation per strata and country of analysis.

We performed the analysis in the countries endemic to lymphatic filariasis at the time of analysis, Myanmar and the Philippines. In Myanmar we estimated a mean difference in district-level deworming coverage between data sources of 43.9% in districts endemic to lymphatic filariasis (districts with any level of lymphatic filariasis treatment programs implemented), and 17.3% in non-endemic districts, although this was limited by the limited number of non-endemic districts (See Appendix). In the Philippines, we estimated a mean difference in district-level deworming coverage between data sources of 22.5% in districts endemic to lymphatic filariasis and 21.9% in non-endemic districts.

*Additional sensitivity analyses*

We varied the data reliability threshold used to exclude WHO reported campaigns. In Burundi, varying the data reliability threshold from excluding campaigns reporting >90% coverage to >100% (not excluding campaigns based on coverage) increased the number of included districts from 13 to 18 out of 18 districts, and increased the mean absolute difference in district-level coverage from 8.7% to 16.7%. In Myanmar, varying the data reliability threshold increased the number of included districts from 2 to 14 out of 15 districts, and the mean absolute difference in district-level coverage from 23.3% to 41.7%. In the Philippines, varying the data reliability threshold increased the number of included districts from 13 to 17 out of 17 districts, and increased the mean absolute difference in district-level coverage from 18.7% to 24.6%.

*Limitations*

The study results should be taken in context with the limitations of the data and assumptions of the analysis. DHS data relied on maternally-reported child deworming receipt, which is subject to recall bias as discussed above. The deworming coverage estimates reported to WHO are estimated using reported pre-school age children populations at risk, although this value is subject to annual fluctuation and inaccuracies in estimation due to within-country migration and measurement concerns. Additionally, as deworming data stratified by child gender were not made available to WHO, no further comparison could be made to gender-stratified coverage estimated by DHS data to assess compatibility for future gender parity analyses. The DHS responses are collected over a 3-9 month period, which may not necessarily overlap with any deworming campaigns, especially in countries with annual deworming or if DHS data are collected during a year with an atypical deworming program implementation. In theory, this could result in under-estimating deworming coverage, although this concern could be mitigated by use of WHO records to identify deworming campaign timelines by country and restricting incorporation of DHS data accordingly.

**Table A1: DHS-VI survey questions in study**

| **Variable** | **Question code** | **Question phrasing** |
| --- | --- | --- |
| Age | HW1 or B19 | Age in months of the child |
| Gender | B4 | Sex of the child |
| District | V101 | De facto region of residence |
| Interview (month) | V006 | Month of interview |
| Interview (year) | V007 | Year of interview |
| Deworming receipt status | H43 | Drugs for intestinal parasites in last 6 months |

**
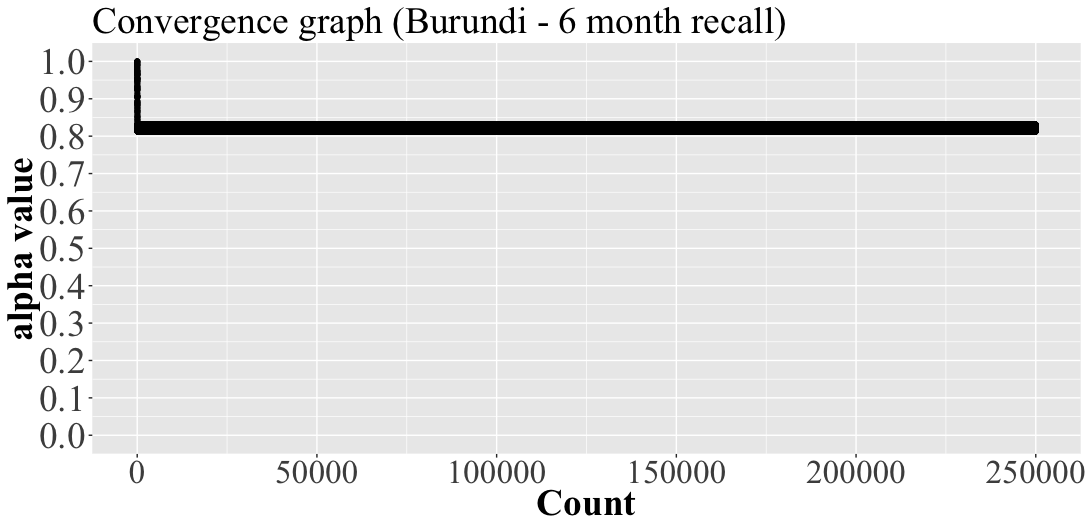
**

**Figure A1: Fitting of “treatment correlation” parameter under base case analysis in Burundi.** We fit the “treatment correlation” parameter used to minimize the sum of square error between coverage estimates reported to WHO and by DHS in regions with overlapping deworming campaigns reported to WHO. The parameter was fit over 250,000 trials.
