# Supplementary material for: Comparison of World Health Organization and Demographic and Health Surveys data to estimate sub-national deworming coverage in pre-school aged children
Source: PLoS Negl Trop Dis. 2020 Aug 17;14(8):e0008551. doi: 10.1371/journal.pntd.0008551 (PMC7462292; doi:10.1371/journal.pntd.0008551)
Supplement: S1 Table — (DOCX) [file pntd.0008551.s003.docx]

**Table S1: District-level deworming coverage in pre-school aged children using data reported to WHO and estimated by DHS in Burundi under base case analysis**

| **District** | **WHO coverage (%)** | **DHS coverage (%), SE** | **Difference in coverage (%)** |
| --- | --- | --- | --- |
| Bubanza | 100.0 | 64.8 ± 2.0 | 35.2 |
| Bujumbura Mairie | 55.1 | 84.5 ± 1.8 | -29.4 |
| Bujumbura Rural | 91.1 | 78.8 ± 1.8 | 12.2 |
| Bururi | 100.0 | 72.4 ± 2.2 | 27.6 |
| Cankuzo | 75.5 | 70.3 ± 2.0 | 5.2 |
| Cibitoke | 90.5 | 58.1 ± 2.0 | 32.4 |
| Gitega | 84.5 | 82.9 ± 1.6 | 1.6 |
| Karusi | 87.1 | 81.8 ± 1.6 | 5.4 |
| Kayanza | 78.9 | 88.6 ± 1.4 | -9.7 |
| Kirundo | 78.1 | 79.0 ± 1.7 | -0.9 |
| Makamba | 100.0 | 70.1 ± 1.9 | 29.9 |
| Muramvya | 88.0 | 81.2 ± 1.8 | 6.8 |
| Muyinga | 81.3 | 64.2 ± 1.9 | 17.1 |
| Mwaro | 85.2 | 84.1 ± 1.7 | 1.1 |
| Ngozi | 75.9 | 77.4 ± 1.8 | -1.5 |
| Rumonge | 100.0 | 74.1 ± 1.9 | 25.9 |
| Rutana | 100.0 | 68.9 ± 2.1 | 31.1 |
| Ruyigi | 74.4 | 73.7 ± 2.1 | 0.7 |

Difference in coverage reported as $\mathrm{Coverage}_{\mathrm{WHO}}-\mathrm{Coverage}_{\mathrm{DHS}}$, (-) indicates coverage estimated by DHS greater than reported to WHO.

SE, standard error
