# Supplementary material for: Comparison of World Health Organization and Demographic and Health Surveys data to estimate sub-national deworming coverage in pre-school aged children
Source: PLoS Negl Trop Dis. 2020 Aug 17;14(8):e0008551. doi: 10.1371/journal.pntd.0008551 (PMC7462292; doi:10.1371/journal.pntd.0008551)
Supplement: S2 Table — (DOCX) [file pntd.0008551.s004.docx]

**Table S2: District-level deworming coverage in pre-school aged children using data reported to WHO and estimated by DHS in Myanmar under base case analysis**

| **District** | **WHO coverage (%)** | **DHS coverage (%), SE** | **Difference in coverage (%)** |
| --- | --- | --- | --- |
| Ayeyarwaddy | 93.5 | 47.0 ± 4.1 | 46.5 |
| Bago | 93.9 | 56.0 ± 4.4 | 37.9 |
| Chin | 93.3 | 56.2 ± 3.9 | 37.1 |
| Kachin | 93.2 | 54.6 ± 4.0 | 38.6 |
| Kayah | 88.4 | 71.1 ± 4.1 | 17.3 |
| Kayin | 94.9 | 54.7 ± 3.7 | 40.2 |
| Magway | 95.9 | 54.2 ± 5.1 | 41.7 |
| Mandalay | 94.9 | 45.2 ± 4.4 | 49.7 |
| Mon | 96.0 | 69.7 ± 4.2 | 26.3 |
| Rakhine | 89.1 | 59.8 ± 4.6 | 29.3 |
| Sagaing | 92.0 | 29.8 ± 4.6 | 62.2 |
| Shan | 95.2 | 36.0 ± 3.8 | 59.2 |
| Taninthayi | 94.1 | 55.4 ± 4.5 | 38.7 |
| Yangon | 94.2 | 35.1 ± 4.5 | 59.1 |

Difference in coverage reported as $\mathrm{Coverage}_{\mathrm{WHO}}-\mathrm{Coverage}_{\mathrm{DHS}}$, (-) indicates coverage estimated by DHS greater than reported to WHO.

SE, standard error
