# Supplementary material for: Comparison of World Health Organization and Demographic and Health Surveys data to estimate sub-national deworming coverage in pre-school aged children
Source: PLoS Negl Trop Dis. 2020 Aug 17;14(8):e0008551. doi: 10.1371/journal.pntd.0008551 (PMC7462292; doi:10.1371/journal.pntd.0008551)
Supplement: S3 Table — (DOCX) [file pntd.0008551.s005.docx]

**Table S3: District-level deworming coverage in pre-school aged children using data reported to WHO and estimated by DHS in the Philippines under base case analysis**

| **District** | **WHO coverage (%)** | **DHS coverage (%), SE** | **Difference in coverage (%)** |
| --- | --- | --- | --- |
| Bangsamoro | 91.4 | 29.9 ± 1.8 | 61.5 |
| Bicol | 84.2 | 60.6 ± 2.1 | 23.5 |
| Cagayan Valley | 85.1 | 65.8 ± 2.6 | 19.3 |
| Calabarzon | 58.3 | 38.6 ± 2.3 | 19.7 |
| Caraga | 85.5 | 71.3 ± 2.0 | 14.2 |
| Central Luzon | 92.9 | 35.6 ± 1.9 | 57.3 |
| Central Visayas | 76.8 | 58.7 ± 2.6 | 18.1 |
| Cordillera | 77.1 | 81.9 ± 1.7 | -4.8 |
| Davao | 97.3 | 73.1 ± 2.1 | 24.2 |
| Eastern Visayas | 70.4 | 71.5 ± 1.9 | -1.1 |
| Ilocos | 79.1 | 56.2 ± 3.1 | 22.9 |
| Mimaropa | 46.8 | 66.3 ± 2.4 | -19.5 |
| National Capital | 77.0 | 24.1 ± 1.8 | 52.9 |
| Northern Mindanao | 88.1 | 72.8 ± 1.9 | 15.3 |
| Soccsksargen | 90.3 | 58.5 ± 2.4 | 31.8 |
| Western Visayas | 70.0 | 49.8 ± 2.1 | 20.2 |
| Zamboanga Peninsula | 42.0 | 53.2 ± 2.8 | -11.2 |

Difference in coverage reported as $\mathrm{Coverage}_{\mathrm{WHO}}-\mathrm{Coverage}_{\mathrm{DHS}}$, (-) indicates coverage estimated by DHS greater than reported to WHO.

SE, standard error
