# Supplementary material for: Comparison of World Health Organization and Demographic and Health Surveys data to estimate sub-national deworming coverage in pre-school aged children
Source: PLoS Negl Trop Dis. 2020 Aug 17;14(8):e0008551. doi: 10.1371/journal.pntd.0008551 (PMC7462292; doi:10.1371/journal.pntd.0008551)
Supplement: S4 Table — (DOCX) [file pntd.0008551.s006.docx]

**Table S4: District-level deworming coverage in children 2-4 years of age using data reported to WHO and estimated by DHS in Myanmar and the Philippines stratified by level of endemicity of lymphatic filariasis (LF)**

| **Country of Analysis** | **Level of LF Endemicity**  **(% sub-districts)** | **Number of Districts** | **Absolute mean difference in coverage (%)** | **Range of difference in coverage (%)** |
| --- | --- | --- | --- | --- |
| Myanmar | 0% | 1 | 8.1 | - |
|  | 1% - 50% | 3 | 35.4 | 19.8 – 56.4 |
|  | 51% - 99% | 1 | 31.7 | - |
|  | 100% | 6 | 38.8 | 25.3 – 54.8 |
|  | < 50% (non-endemic) | 3 | 19.3 | 8.1 – 29.9 |
|  | ≥ 50% (endemic) | 8 | 40.1 | 25.3 – 56.4 |
|  | 0% (non-endemic) | 1 | 8.1 | - |
|  | > 0% (endemic) | 10 | 37.1 | 19.8 – 56.4 |
| Philippines | 0% (non-endemic) | 10 | 21.9 | -26.1 – 58.5 |
|  | > 0% (endemic) | 6 | 22.5 | -18.0 – 58.5 |

Difference in coverage reported as $\mathrm{Coverage}_{\mathrm{WHO}}-\mathrm{Coverage}_{\mathrm{DHS}}$, (-) indicates coverage estimated by DHS greater than reported to WHO.
