# Supplementary material for: Comparison of World Health Organization and Demographic and Health Surveys data to estimate sub-national deworming coverage in pre-school aged children
Source: PLoS Negl Trop Dis. 2020 Aug 17;14(8):e0008551. doi: 10.1371/journal.pntd.0008551 (PMC7462292; doi:10.1371/journal.pntd.0008551)
Supplement: S1 Fig — We estimated the sample size of DHS respondents at the district-level and estimated the proportion eligible for one or two deworming campaigns based on a maternal recall period of 6 months. This data is visualized for Burundi (panel A-B), Myanmar (panel C-D), and the Philippines (panel E-F), each with a varying maternal recall period from 4 or 8 months. (DOCX) [file pntd.0008551.s007.docx]

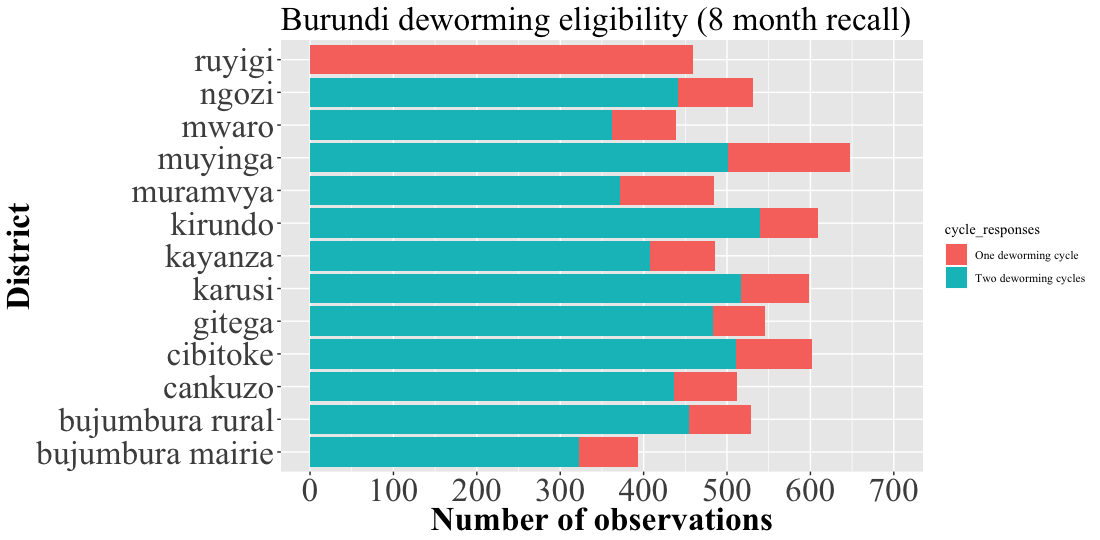

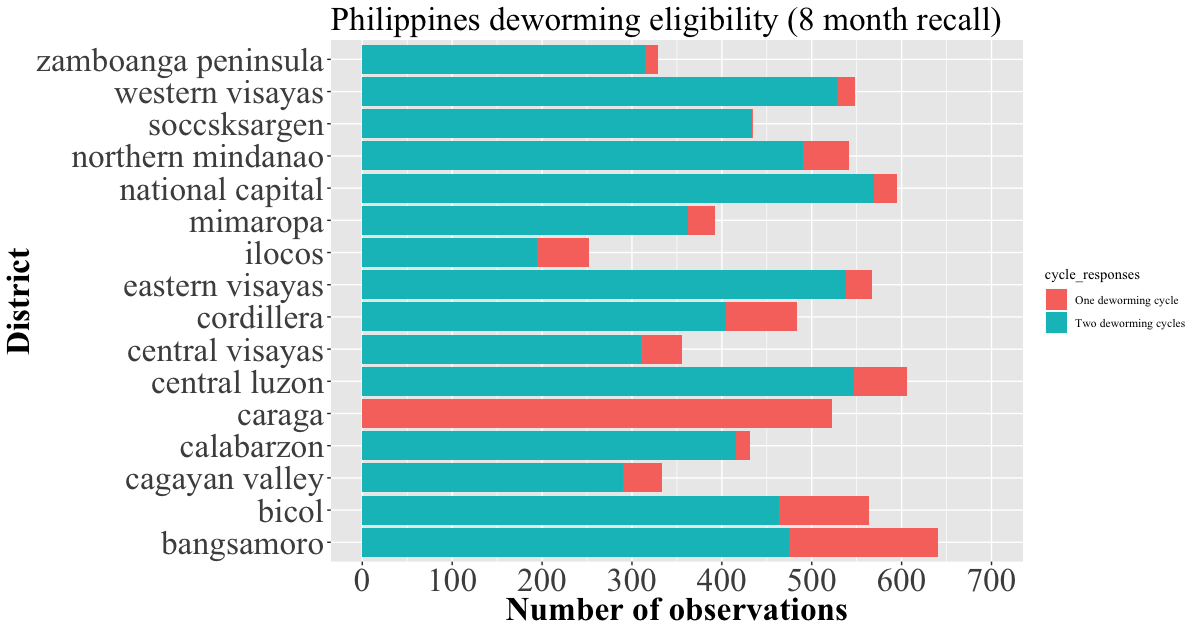

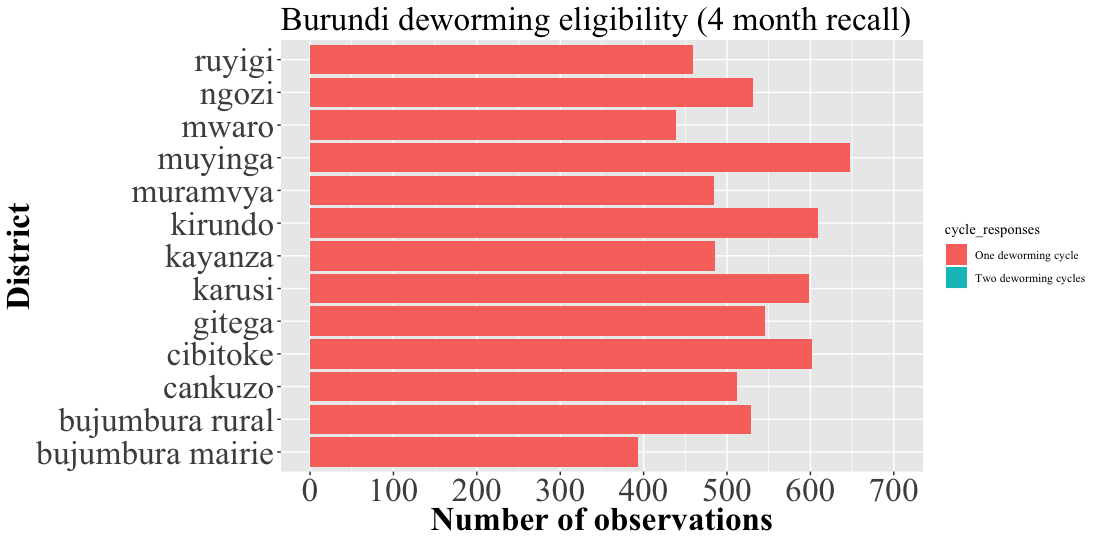

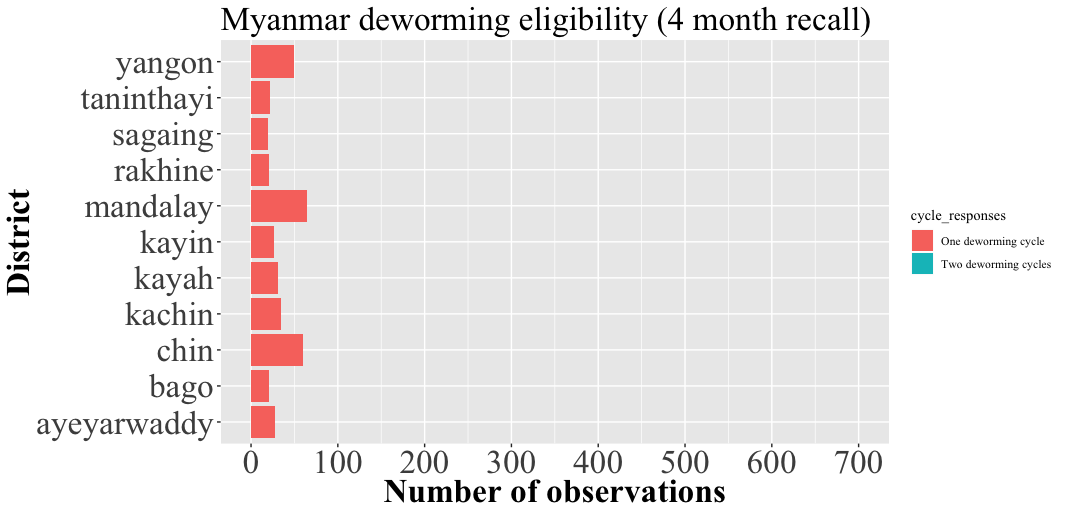

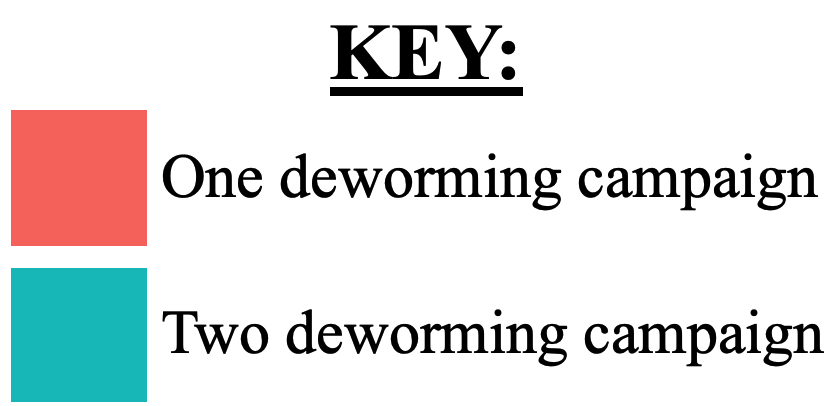


**A**


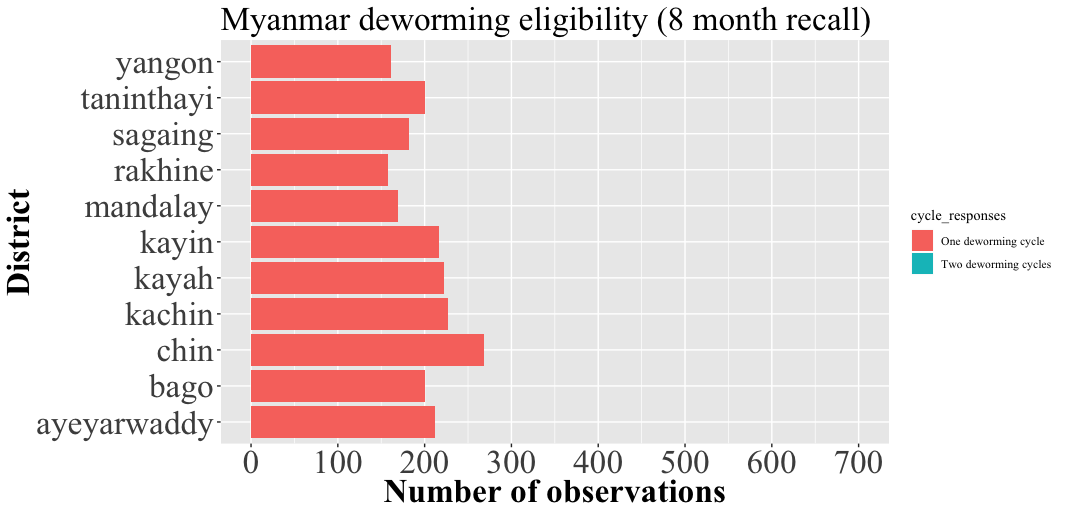

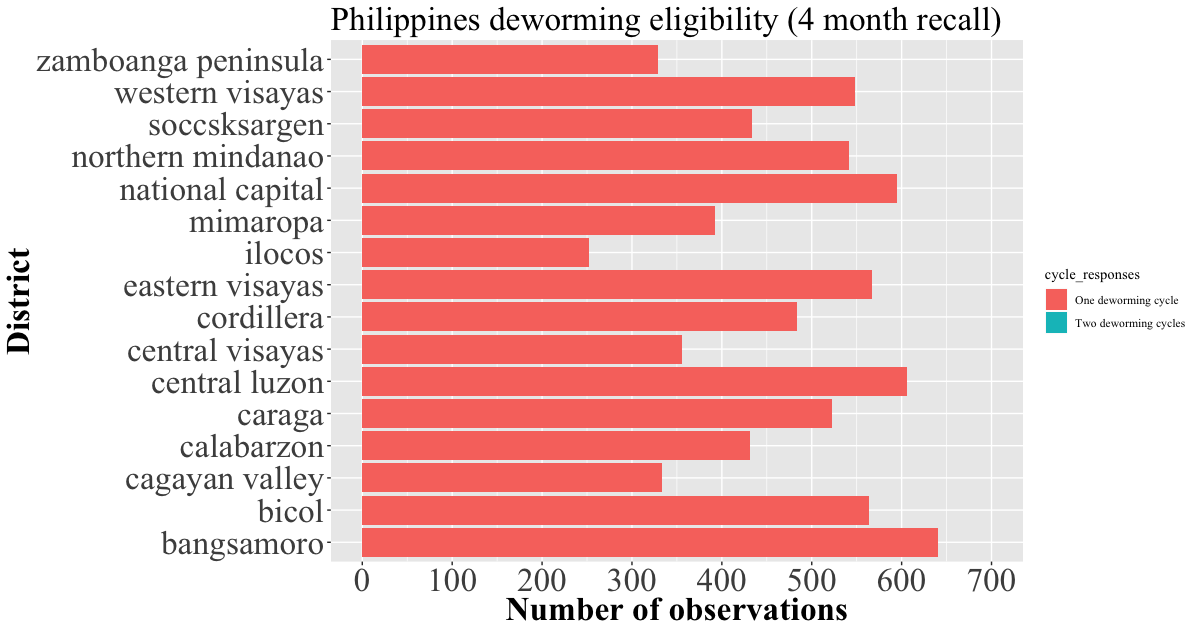


**F**

**BD**

**D**

**E**

**C**

**Figure S1: District-level sample size of DHS observations used to estimate deworming coverage in study countries, varying maternal recall period.** We estimated the sample size of DHS respondents at the district-level and estimated the proportion eligible for one or two deworming campaigns based on a maternal recall period of 6 months. This data is visualized for Burundi (panel A-B), Myanmar (panel C-D), and the Philippines (panel E-F), each with a varying maternal recall period from 4 or 8 months.
