# Supplementary material for: Comparison of World Health Organization and Demographic and Health Surveys data to estimate sub-national deworming coverage in pre-school aged children
Source: PLoS Negl Trop Dis. 2020 Aug 17;14(8):e0008551. doi: 10.1371/journal.pntd.0008551 (PMC7462292; doi:10.1371/journal.pntd.0008551)
Supplement: S2 Fig — We obtained WHO data on district-level deworming coverage reported by national Ministries of Health based on health facility records. We estimated district-level deworming coverage with DHS data corresponding to the known WHO deworming campaigns using maternally-reported data on child deworming receipt. The comparison of deworming coverage using WHO and DHS data are shown for the base case recall period of 6-month in Burundi (panel A), Myanmar (panel B) and the Philippines (panel C) based on the DHS question formulation. (DOCX) [file pntd.0008551.s008.docx]

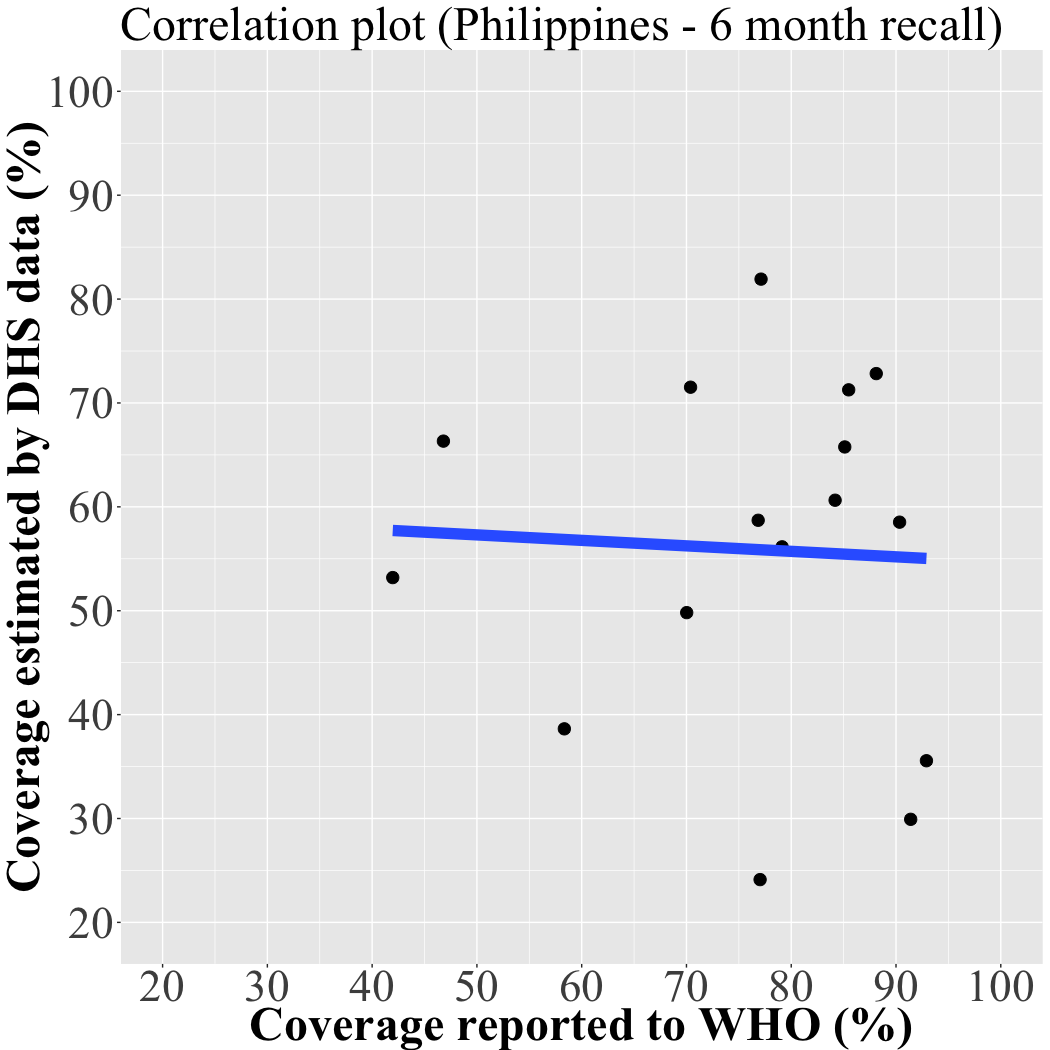

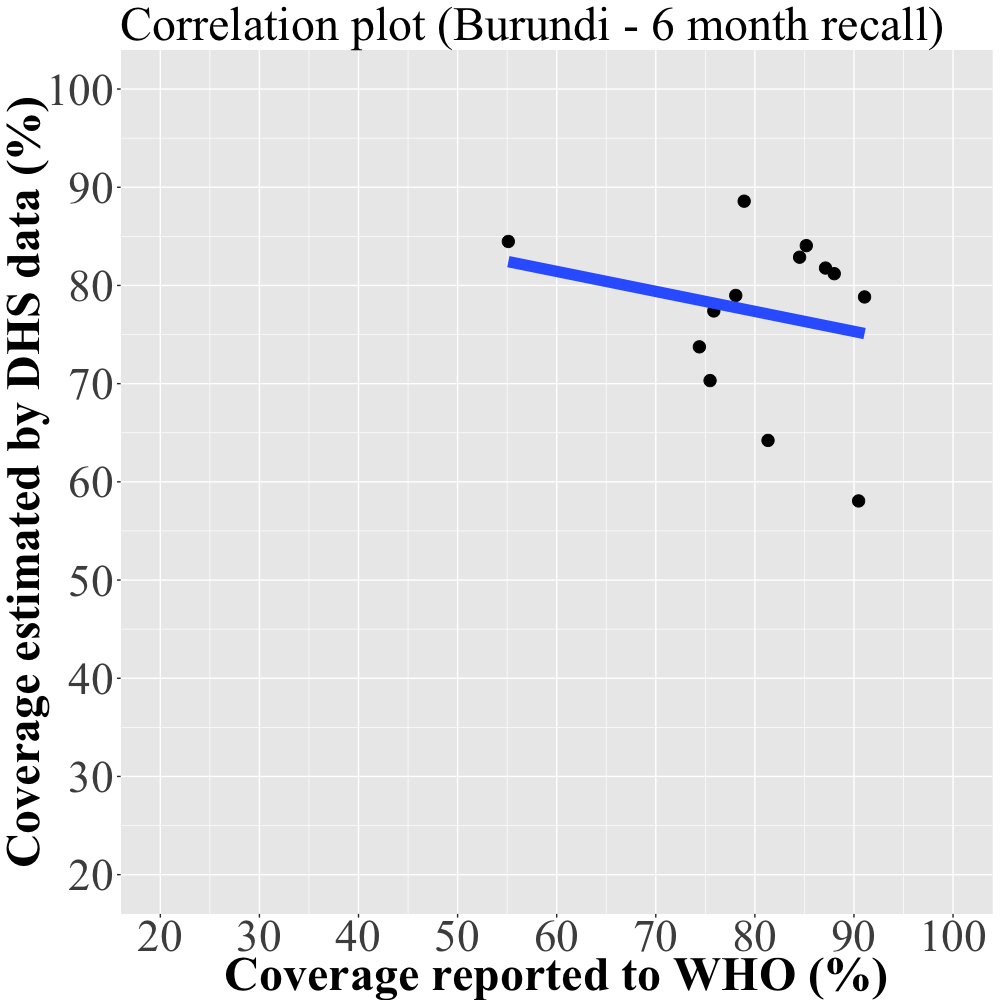

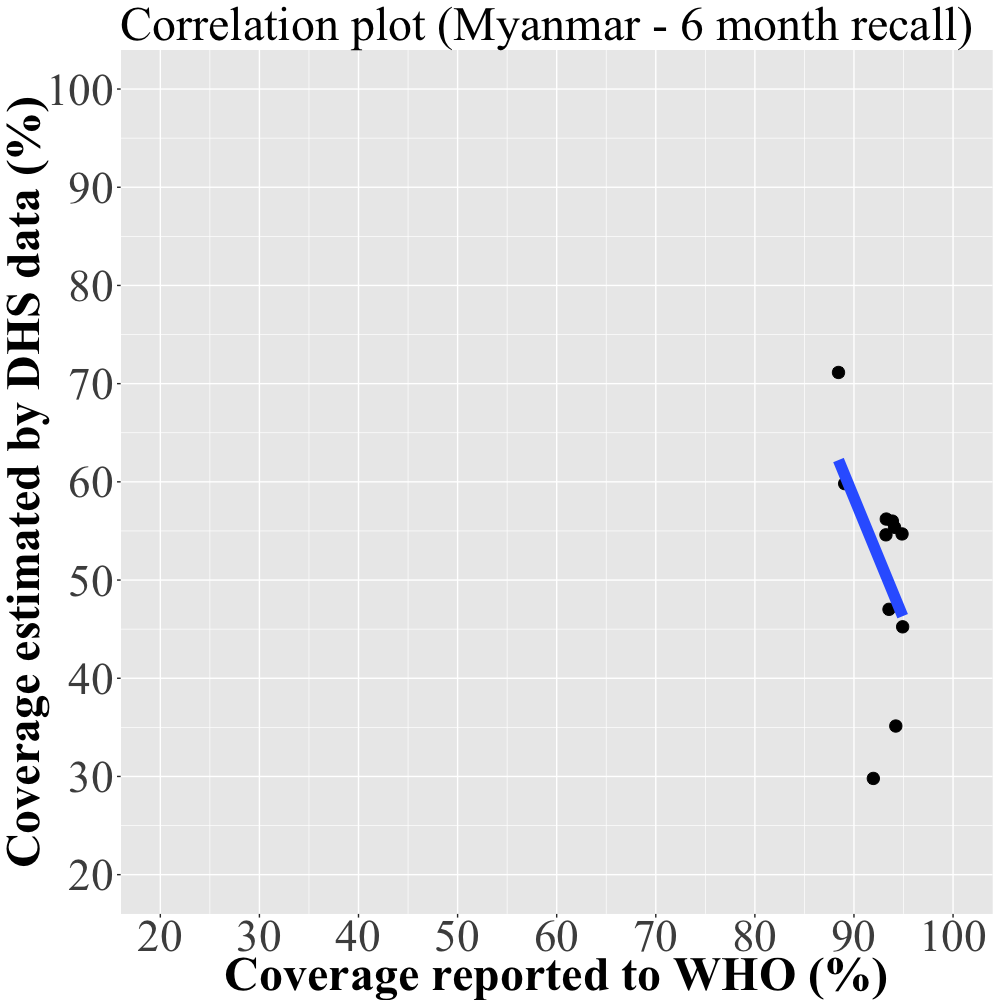


**A**

**CDC**

**B**

**Figure S2. Correlation between district-level deworming coverage in pre-school aged children reported to WHO and estimated using DHS data for study countries.** We obtained WHO data on district-level deworming coverage reported by national Ministries of Health based on health facility records. We estimated district-level deworming coverage with DHS data corresponding to the known WHO deworming campaigns using maternally-reported data on child deworming receipt. The comparison of deworming coverage using WHO and DHS data are shown for the base case recall period of 6-month in Burundi (panel A), Myanmar (panel B) and the Philippines (panel C) based on the DHS question formulation.
