# Supplementary material for: Comparison of World Health Organization and Demographic and Health Surveys data to estimate sub-national deworming coverage in pre-school aged children
Source: PLoS Negl Trop Dis. 2020 Aug 17;14(8):e0008551. doi: 10.1371/journal.pntd.0008551 (PMC7462292; doi:10.1371/journal.pntd.0008551)
Supplement: S4 Fig — We obtained WHO data on district-level deworming coverage reported by national Ministries of Health based on health facility records. We estimated district-level deworming coverage with DHS data corresponding to the known WHO deworming campaigns using maternal-reported data on child deworming receipt. The comparison of deworming coverage using WHO and DHS data is shown with a varying data reliability threshold (base case of 95%) from > 90% to no threshold to change the districts meeting inclusion criteria in Burundi (panel A-B), Myanmar (panel C-D), and the Philippines (panel E-F). (DOCX) [file pntd.0008551.s010.docx]

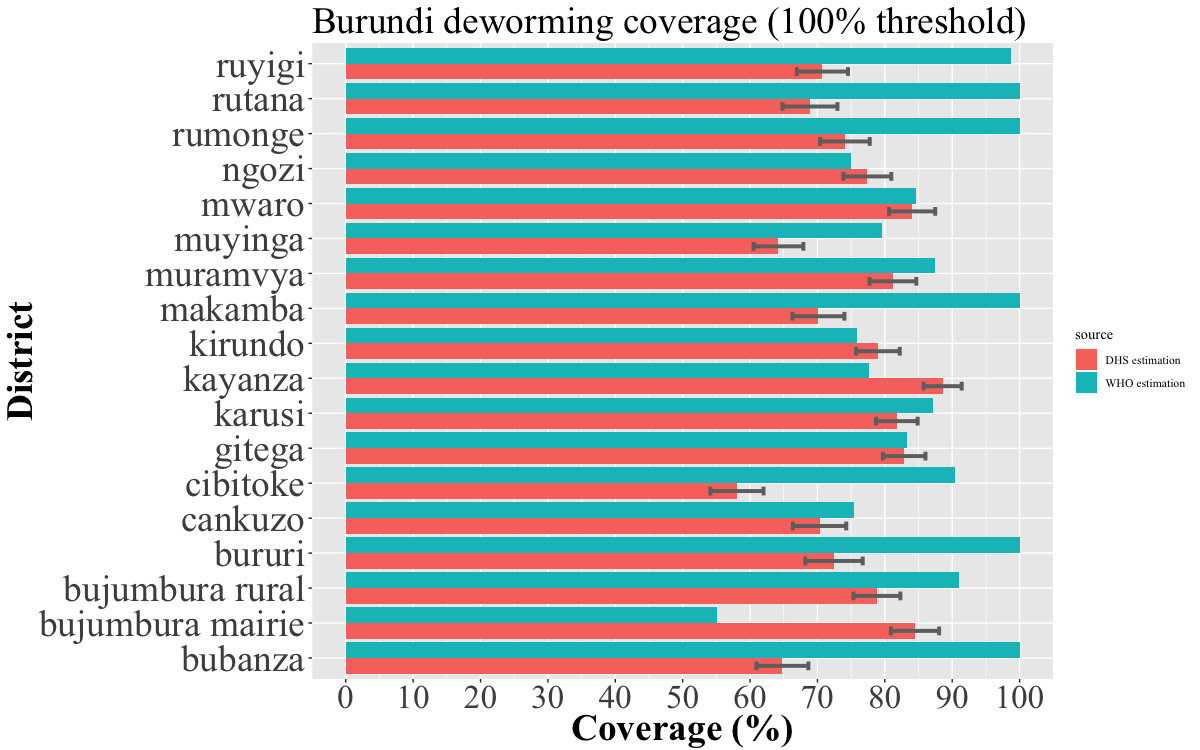

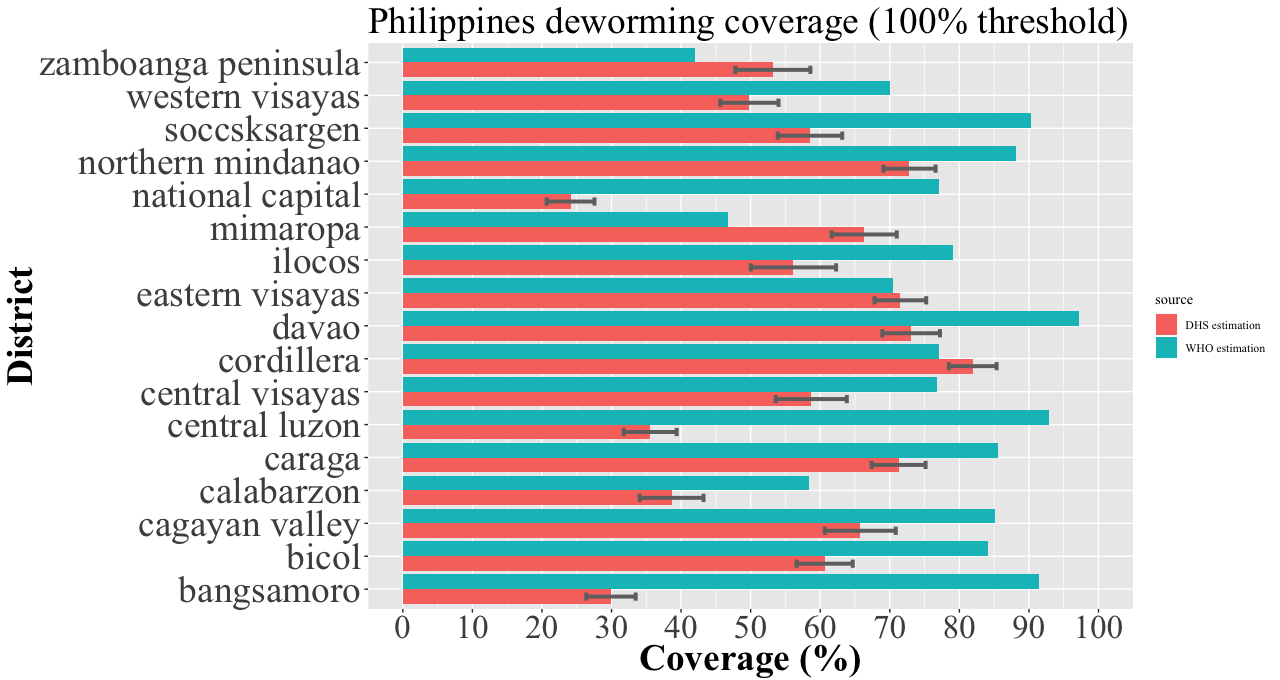

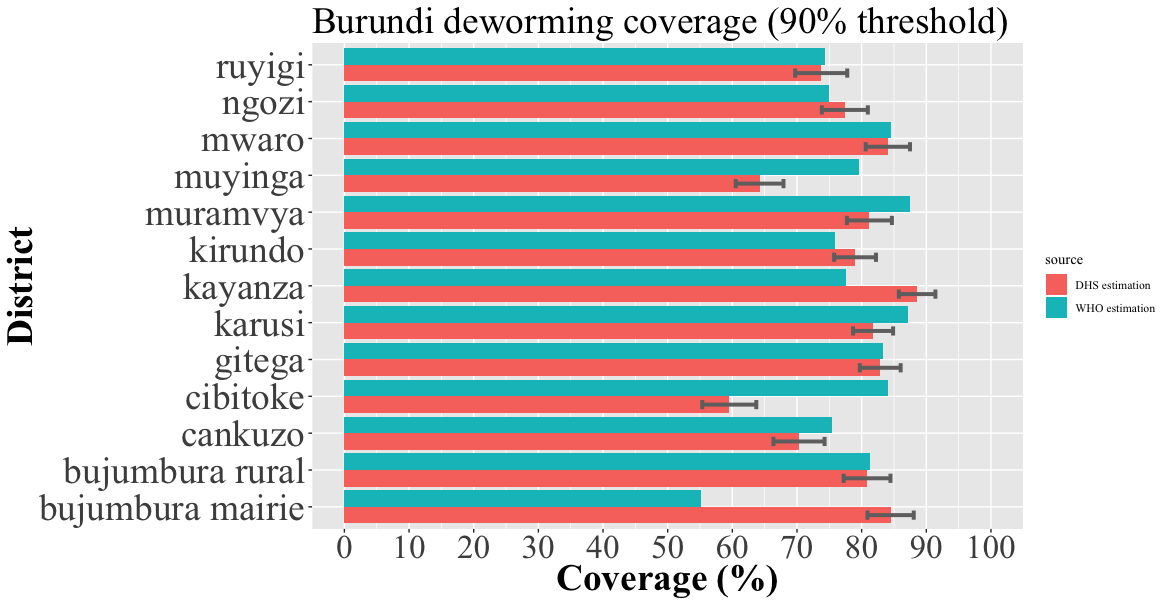

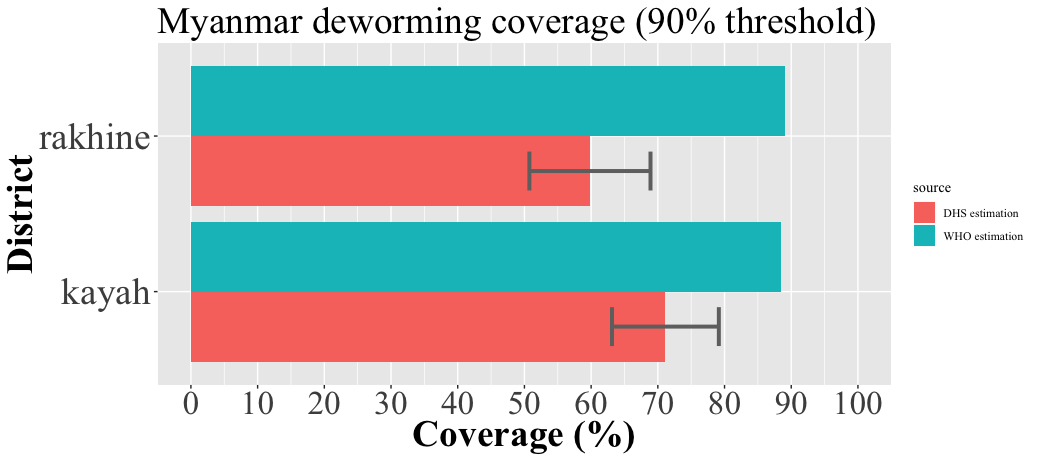

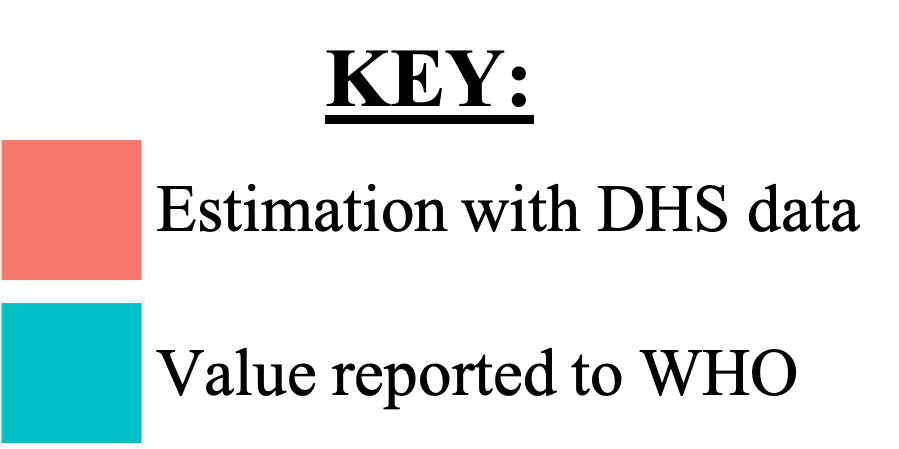


**A**


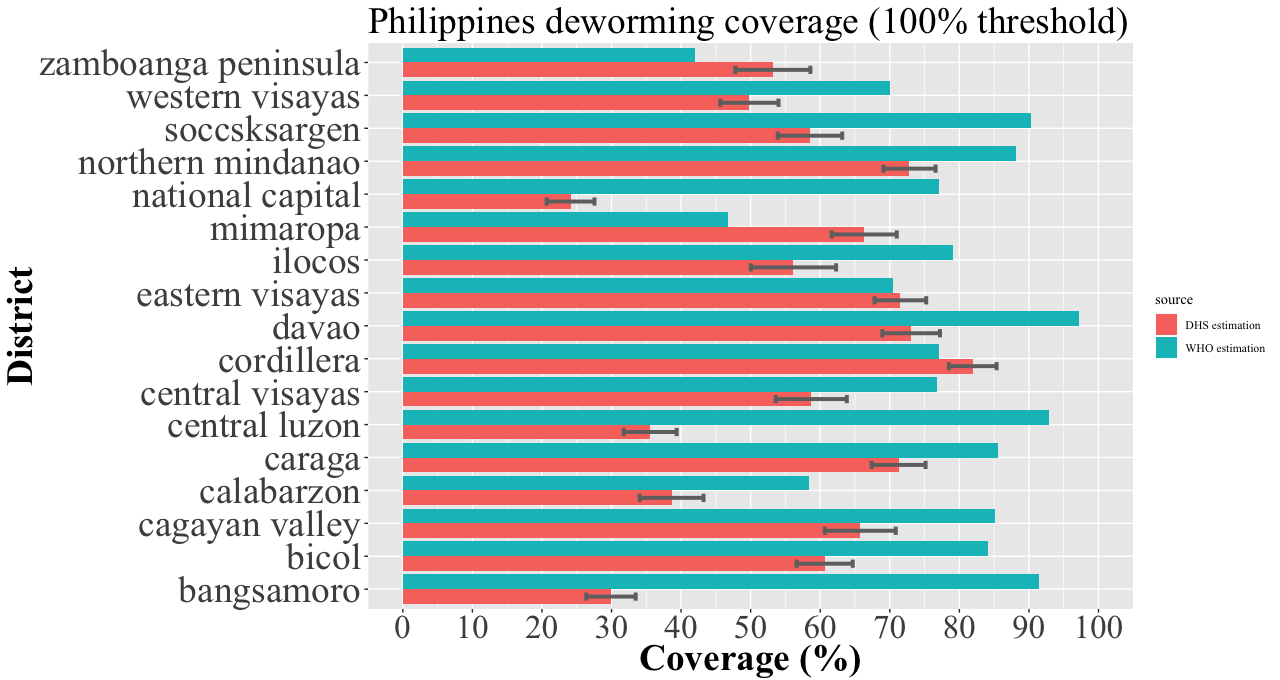

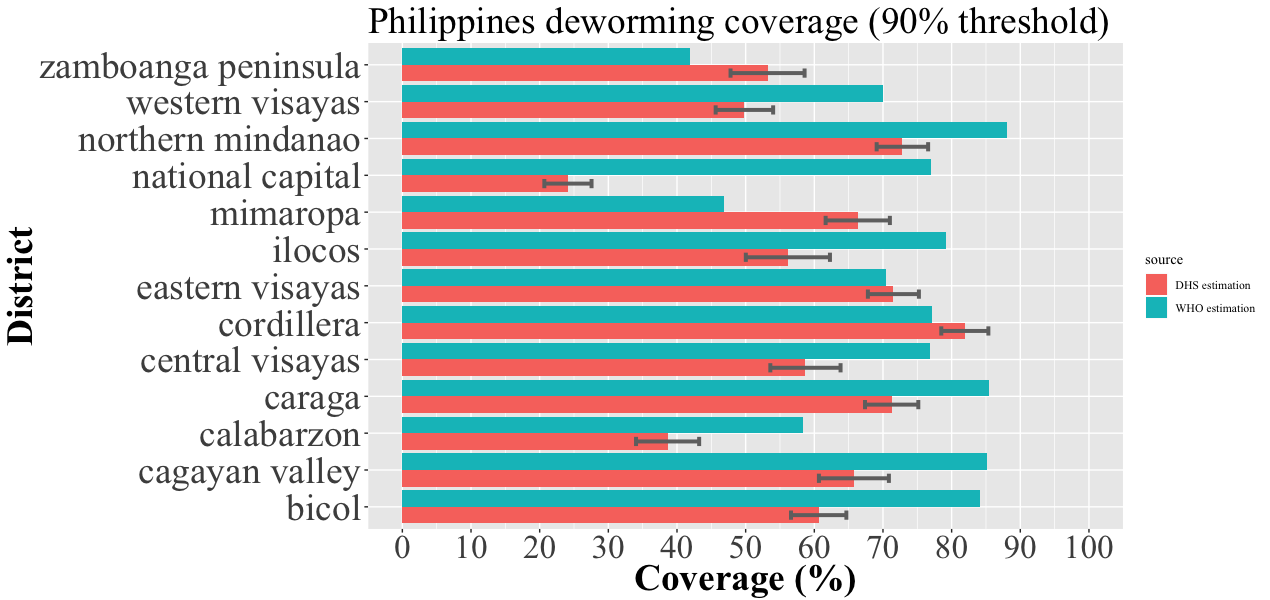


**F**

**BD**

**D**

**E**

**C**

**Figure S4: Comparison of district-level deworming coverage in pre-school aged children reported to WHO and estimated by DHS in study countries, varying threshold for data reliability.** We obtained WHO data on district-level deworming coverage reported by national Ministries of Health based on health facility records. We estimated district-level deworming coverage with DHS data corresponding to the known WHO deworming campaigns using maternal-reported data on child deworming receipt. The comparison of deworming coverage using WHO and DHS data is shown with a varying data reliability threshold (base case of 95%) from > 90% to no threshold to change the districts meeting inclusion criteria in Burundi (panel A-B), Myanmar (panel C-D), and the Philippines (panel E-F).
