# Supplementary material for: Comparison of World Health Organization and Demographic and Health Surveys data to estimate sub-national deworming coverage in pre-school aged children
Source: PLoS Negl Trop Dis. 2020 Aug 17;14(8):e0008551. doi: 10.1371/journal.pntd.0008551 (PMC7462292; doi:10.1371/journal.pntd.0008551)
Supplement: S7 Fig — Endemicity to lymphatic filariasis was determined by sub-district-level data reported to WHO on the record of a treatment campaign during the year of deworming analysis within each country. In Myanmar, districts were categorized as endemic to lymphatic filariasis if half of sub-districts received treatment (panel A) and in the Philippines, districts were categorized as endemic to lymphatic filariasis if any of their sub-districts received treatment (panel B). Note difference in endemicity definition used for Myanmar and the Philippines. (DOCX) [file pntd.0008551.s013.docx]

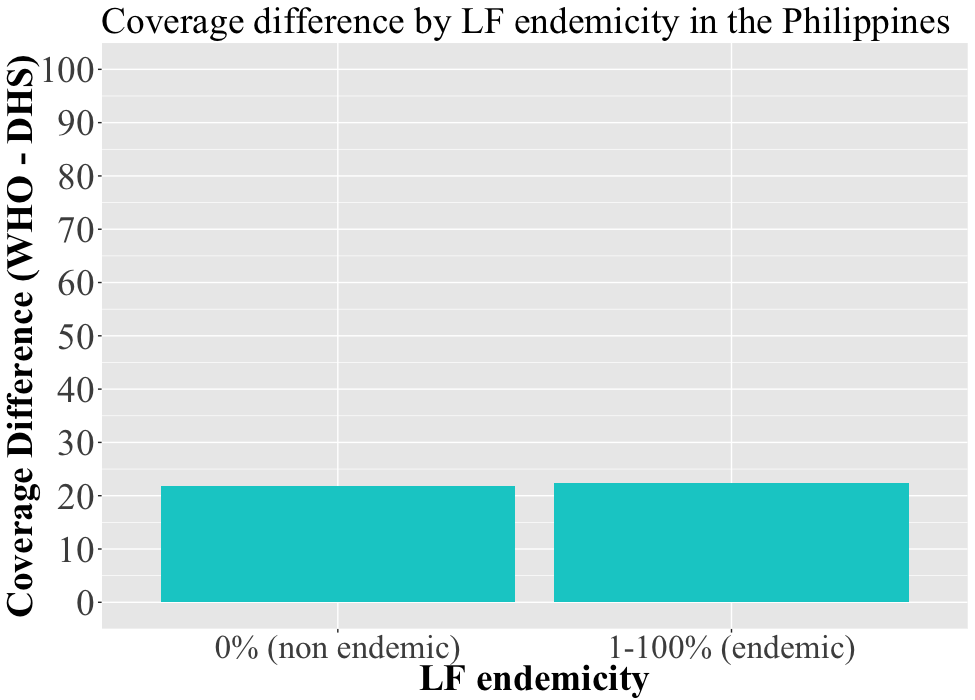

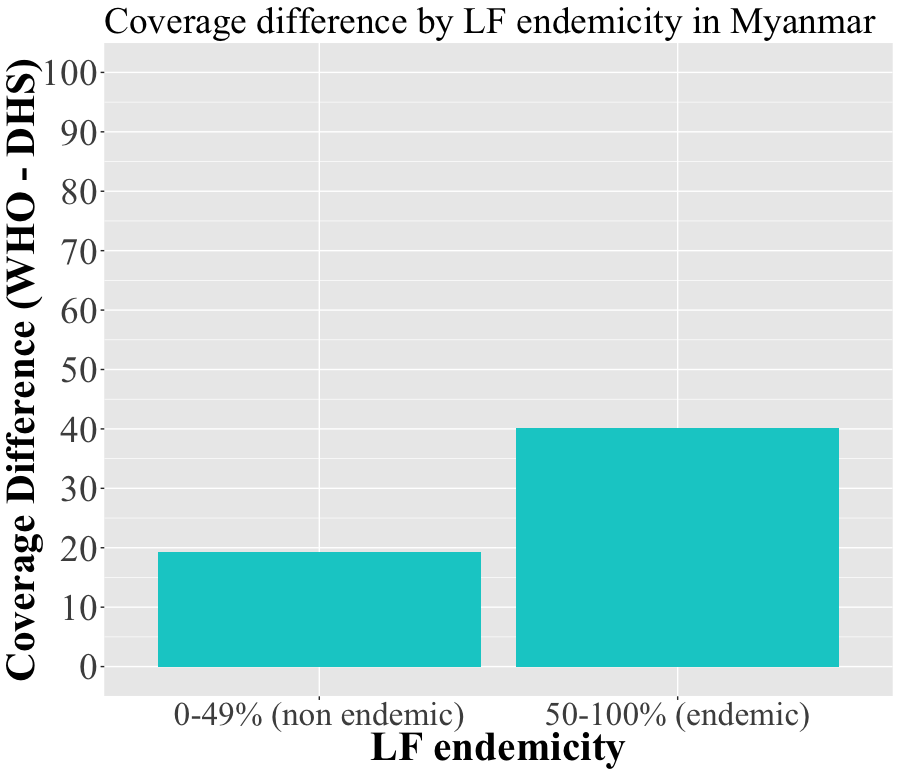


**A**

**BD**

**Figure S7: Comparison of mean district-level deworming coverage in children 2-4 years of age reported to WHO and estimated by DHS for countries endemic to lymphatic filariasis stratified by level of endemicity.** Endemicity to lymphatic filariasis was determined by sub-district-level data reported to WHO on the record of a treatment campaign during the year of deworming analysis within each country. In Myanmar, districts were categorized as endemic to lymphatic filariasis if half of sub-districts received treatment (panel A) and in the Philippines, districts were categorized as endemic to lymphatic filariasis if any of their sub-districts received treatment (panel B). Note difference in endemicity definition used for Myanmar and the Philippines.
