# Supplementary material for: Comparison of World Health Organization and Demographic and Health Surveys data to estimate sub-national deworming coverage in pre-school aged children
Source: PLoS Negl Trop Dis. 2020 Aug 17;14(8):e0008551. doi: 10.1371/journal.pntd.0008551 (PMC7462292; doi:10.1371/journal.pntd.0008551)
Supplement: Attachment — Submitted filename: 2020 PNTD Response to Editor- Lo et al.docx [file pntd.0008551.s014.docx]

“Comparison of World Health Organization and Demographic and Health Surveys data to estimate sub-national deworming coverage in pre-school aged children” (reference no. PNTD-D-20-00061) – Point-by-point response

20 April 2020

Dear Editor,

We are pleased to submit a revised version of our Research Article, entitled “Comparison of World Health Organization and Demographic and Health Surveys data to estimate sub-national deworming coverage in pre-school aged children” (reference no. PNTD-D-20-00061). We thank you and the two external reviewers very much for the careful examination and helpful comments, and for the invitation to submit a revised manuscript. We have addressed the issues brought forward. In particular, we focused on adding clarity to many of the terms used in our methodologies and broadening our discussion on the applications of the analyses presented. Below, please find our point-by-point response, clearly indicating the changes made in the manuscript.

We believe this article is timely given the global push to achieve the approaching coverage goals set by the 2020 WHO Roadmap. We trust that our findings will be of interest to the readership of PLOS Neglected Tropical Diseases, and will support efforts to improve data accuracy and to develop novel metrics to assess geographic variation and equity in coverage globally.

Sincerely,

Nathan C. Lo, MD PhD

University of California, San Francisco

On behalf of co-authors

**Response to Reviewer #1**

**General Comments**

*Comment #1*

General: this is a useful, relevant paper for advancing determination of deworming coverage.

Response: We thank Reviewer #1 for their kind words and helpful comments.

*Comment #2*

Abstract: Would state explicitly what time frame national deworming coverage reported to WHO is intended to represent (ideally would be the past 6 months, for comparability to the maternal report question, but regardless readers will need some sense of how these two sources would be compared).

Response: We appreciate the opportunity to clarify this important point. The data reported to WHO is aggregated for each mass drug administration campaign, with date and coverage reported. The data spans over multiple years as outlined in the methods. We have added additional details to the methodology section to clarify our assumptions for the timeframe of data reported to WHO.

-In Abstract:

“**WHO provided data on the date and sub-national coverage per mass drug administration** reported **to** Ministries of Health. DHS included maternally-reported deworming status within the past 6 months for each child surveyed.”

-In Methods:

“Coverage reported to WHO was computed as the total number of individuals reported to have received deworming medications over the total population of targeted pre-school aged children per district for a single deworming campaign with adjustments to be aligned with DHS data (30). **We assume all deworming medications reported to WHO for a deworming campaign were received within the month of deworming campaign initiation.**”

*Comment #3*

Introduction: Optionally, authors may also wish to consider mentioning the neurodevelopmental effects of heavy STH infestation.

Response: We thank the reviewer for this suggestion and have updated the manuscript to also discuss the cognitive/neurological imparities attributed to heavy infection.

-In Introduction:

“The burden of STH is associated with a range of clinical morbidities including abdominal pain, anemia, malnutrition, and **in cases of heavy STH infection, physical (e.g. growth) and possible developmental effects**, some of which may be addressed through treatment with albendazole or mebendazole (1,6–9).”

-In References:

“9.	Gall S, Müller I, Walter C, Seelig H, Steenkamp L, Pühse U, et al. Associations between selective attention and soil-transmitted helminth infections, socioeconomic status, and physical fitness in disadvantaged children in Port Elizabeth, South Africa: An observational study. PLoS Negl Trop Dis. 2017 May;11(5):e0005573.”

*Comment #4*

Introduction: The basic issue authors are raising in the NTD world, of conflict between official coverage figures based on dose numbers administered (“administrative coverage”) and population-based survey results, has a long history in the world of immunization as well. Authors may find it useful to explore this and optionally mention parallels, since there is vast experience in that field with the issue. One link is https://urldefense.proofpoint.com/v2/url?u=https-3A__www.who.int_immunization_monitoring-5Fsurveillance_routine_coverage_en_&d=DwIGaQ&c=iORugZls2LlYyCAZRB3XLg&r=zpCqyccb0GcHlNnAbDg2Fz698zmK uNMhCQQqgwQ6hI&m=flsrInU5f54EEvX1DYl7TFLGkmxU8VVqDDcNw9bQ3sY&s=j900kPc3MhsATYUmurAypnNd1W6I3S3Dw3bNPs6rf3I&e= . Also, the discussion of the advantages and disadvantages of each data source found here <https://www.who.int/bulletin/volumes/87/7/08-053819/en/> may be useful.

Response: We appreciate the reviewer’s attention to these very relevant resources. We have expanded our discussion section to draw parallels between our proposed deworming data comparisons with those discussed in the vaccination literature.

-In Discussion:

“The deworming coverage data reported to WHO and estimated by DHS measure deworming using different mechanisms. Most notably, DHS deworming data are reported based on maternal recall and may include coverage by unprogrammed sources (e.g. local pharmacy, clinic) and albendazole receipt for non-STH related public health campaigns (e.g. as part of lymphatic filariasis treatment) in addition to programmed sources (i.e., national STH deworming programs). As DHS data relied on maternally-reported child deworming receipt, it is potentially subject to misclassification of treatment arising from recall bias. However, recent work suggests maternally-reported child deworming data are a reliable estimate of programmatic coverage in the short-term period following medication, with reported estimates within 5-10% of official campaign estimates **and with vaccine coverage estimates by WHO routinely incorporating maternally-reported survey data** (39,40). In aggregating data at multiple points from individual health facility records up to the national-level, it is possible that records reported to WHO were re-counted or contained errors, which may both have over- or under-estimated coverage reported; however this is not possible for DHS datasets which provided household level survey data. **Similar differences in data collection methodologies have been noted in the vaccine literature when comparing annual administrative and survey data on vaccine coverage. As both sources have their merits and limitations, agencies often rely on field experts to determine the most probable vaccine coverage with WHO recommendations for the periodic implementation of data quality and coverage evaluation surveys (40-42).**”

-In References

“40.	World Health Organization. Immunization coverage [Internet]. World Health Organization; 2020. Available from: https://www.who.int/immunization/monitoring_surveillance/routine/coverage/en/

41.	Burton A. WHO and UNICEF estimates of national infant immunization coverage: methods and processes. Bull World Health Org. 2009 Jul 1;87(7):535–41.”

*Comment #5*

Introduction: Use of Terms

a) Deworming is not a strategy for ‘eliminating’ the disease burden of STH—‘elimination’ has specific public health definitions which I do not believe is the goal of these programs. Perhaps ‘controlling’ / ‘minimizing’?

Response: We have updated the introduction to reflect the language used in previously published deworming literature.

-In Introduction:

“Preventive chemotherapy, which involves the periodic, coordinated, large-scale administration of single-dose albendazole or mebendazole (i.e., deworming treatment) to groups at risk, is the primary public health strategy to **control** STH burden by treating prior infections and decreasing worm burden to prevent future morbidity (4,10).”

b) Mass chemotherapy is not preventive – it’s intended to treat existing infestations in individuals.

Response: We appreciate the reviewers comment and have further clarified use of the term “preventive chemotherapy” to explain to readers outside the field.

-In Introduction:

“Preventive chemotherapy, which involves the periodic, coordinated, large-scale administration of single-dose albendazole or mebendazole (i.e., deworming treatment) to groups at risk, is the primary public health strategy to **control** STH burden **by treating prior infections and decreasing worm burden to prevent future morbidity** (4,10).”

-In References

“10.	World Health Organization, Nutrition for Health and Development, World Health Organization, Department of Control of Neglected Tropical Diseases, World Health Organization. Preventive chemotherapy to control soil-transmitted helminth infections in at-risk population groups: guideline. [Internet]. 2017 [cited 2020 Mar 26]. Available from: http://www.ncbi.nlm.nih.gov/books/NBK487927/”

c) WHO does not have an ongoing goal to achieve national coverage at any threshold in ‘all countries’ - please check WHO deworming recommendations. Probably ‘all endemic countries’ or ‘all highly endemic countries’ etc – maybe all countries above the baseline 20% prevalence threshold in the link that follows. Also, it may be important to clarify here that WHO recommendations for frequency of deworming (annual vs biannual) depend on the baseline prevalence <https://www.who.int/elena/titles/full_recommendations/deworming/en/>

Response: We appreciate the reviewer’s attention to detail and agree the 2020 WHO goals only apply to countries where STH is endemic in any district. We have updated the manuscript to reflect this clarification.

-In Introduction:

“The ongoing public health goal set against STH by WHO is to achieve a minimum of 75% annual national coverage in both school-aged and pre-school aged children by 2020 for all countries **where STH remains endemic** (4,10).”

*Comment #6*

Introduction: Introduction should please make clear what this analysis provides that has not been done before. Have other studies sought to compare coverage from these two data sources, or other similar data sources, in other countries? Is this the first to do it on a subnational level? If, as implied in your discussion, this is the first attempt to triangulate using data already routinely collected for a different purpose rather than a purpose-built deworming coverage survey and you think its primary value is in the potential resources saved, would say that.

Response: We thank the reviewer for their suggestion. We have updated the manuscript to reflect the manners in which our study may lend novel insights, specifically focusing on how this is the first study to evaluate sub-national coverage reported to WHO and also the first to use a routinely collected data source.

-In Introduction:

“**Although previous analyses have compared coverage reported to WHO at the national-level with coverage estimated from specialized validation surveys conducted by external public health institutes, no studies have evaluated the use of sub-national coverage (27,28).** The DHS are a potential source of routinely collected, sub-national deworming data to compare coverage data from existing deworming program evaluation efforts, although questions remain as to how these coverage estimates will compare to data reported to WHO.”

**Methods Comments**

*Comment #7*

Please clarify in the manuscript the inclusion criteria for DHS-campaign dataset ‘pairs’.

a) Were campaigns reported to WHO included only if a DHS took place in the same district within 6 months after the campaign? Were DHSs included only if a campaign had taken place in the same district within the previous 6 months? Could a given district be included more than once if it had two different DHSs, each within six months of a different deworming campaign?

Response: We appreciate the reviewers comment and have updated the methodologies surrounding how WHO data and DHS surveys were paired. We have clarified the methods section as below.

-In Methods:

“The coverage data were matched geospatially (at district-level) and temporally (aligning DHS administration dates within the recall period [time period where observations collected likely refer to a source of programmatic coverage; 6 months in the DHS] following campaigns reported to WHO). **This produced a pair of coverage estimates (one DHS estimated and one reported to WHO) only for districts where 1) a campaign was reported to WHO and 2) any DHS responses were collected within the recall period following the campaign reported to WHO.**”

“The primary study outcome was reported deworming receipt by data source; we estimated **pairs of deworming** coverages and **the** mean absolute difference in district-level coverage reported to WHO and estimated by DHS **only for districts with both DHS data and data reported to WHO available.**”

“As surveys were conducted over an extended period, we also excluded **individual** DHS observations **with their** individual survey administration date outside the recall period **following** any campaign reported to WHO for their district of residence **as deworming captured by these excluded observations could not refer to a known source of programmatic coverage.**”

“First, for each deworming campaign reported to WHO, we determine if any children in the DHS dataset were eligible to have been exposed based on their unique administration date. **Any deworming campaigns with no eligible DHS observations were excluded from data reported to WHO.**”

“For districts where all children in DHS data were eligible for only one deworming campaign reported to WHO based on each child’s survey administration date uniquely overlapping with one campaign recall period, we directly used the corresponding campaign coverage as the treatment probability for each child targeted for treatment. **For districts where some children in DHS data were** eligible for two deworming campaigns reported to WHO, based on the overlap of DHS administration dates with the recall periods of multiple deworming campaigns reported to WHO, we used a “treatment correlation” parameter, α, to combine coverage reported to WHO from all applicable campaigns into a single treatment probability for all children targeted for treatment (See Appendix). **This ensured, at maximum, we generated one coverage estimate reported to WHO for each district, regardless of the number of campaigns that met inclusion criteria.**”

b) What does a ‘base’ of 6 months that varied from 4-8 months mean? – what criteria were actually applied to yield this range?

Response: We appreciate the opportunity to further clarify. The default maternal recall in the DHS survey is 6 months as specified in the deworming survey question. However, due to likely imperfect recall, whether using a shorter period (4 months) or longer period (8 months), we vary this as a sensitivity analysis.

-In Methods:

“We conducted a series of sensitivity analyses on key parameters and assumptions to determine the robustness of our findings. **In one such analysis, we adjusted DHS survey inclusion by varying** the recall period assumed for maternally-reported response (base case of 6 months) **across a short-term period of 4 to 8 months that we believed would still yield reliable DHS responses to child deworming receipt to evaluate cases when maternal recall was shorter or longer than designated by the original** **question.**”

c) Since DHSs are not done instantly, did the time windows for inclusion correspond to the beginning or the end of the DHS data collection period in the district? What if a DHS was started in the district less than 6 months after a campaign, but finished more than 6 months?

Response: We appreciate the reviewer’s comment and have updated the manuscript to clarify that each DHS observation has a unique administration date which was used to determine recall eligibility rather than using a district-level administration date. With this approach, we were able to account for the time window of survey administration in our analysis.

-In Methods:

“**As surveys were conducted over an extended period, we** also excluded **individual** DHS observations with **their individual** survey administration date outside the recall period **following** any campaign reported to WHO for their district of residence as deworming captured by these excluded observations could not refer to a known source of programmatic coverage.”

d) Related, what does ‘assumed recall’ of 6 months in the DHS mean? If it refers to the time period specifically listed in the DHS question asked of the respondent, there’s nothing ‘assumed’ about it. I’d recommend getting rid of or defining the term. If the length of time respondents were asked about varied by country, I would state that clearly when you quote the DHS question, and since there were just three countries, perhaps state which it was for each. Later the term ‘recall period’ recurs – similarly please define this if you need to keep it. It’s not clear to me that there’s a role here for worrying about the length of a respondent’s recall at all since the time window is clearly defined.

Response: We appreciate the reviewer’s comment and agree a definition for “recall period” would add clarity to the methodology. We elect to retain the term as we are working with survey data which may be subject to recall biases potentially arising from poor recall of the exact month of child deworming and therefore vary the recall period in our sensitivity analyses. We have expanded the methods section to include a definition for “recall period”, an expanded explanation for its use as an exclusion criterion, and the rationale for varying the recall period in the sensitivity analysis. Also see additional explanation in comment #7b.

-In Methods:

“The coverage data were matched geospatially (at district-level) and temporally (aligning DHS administration dates within the recall period [**time period where observations collected likely refer to a source of programmatic coverage;** 6 months **in the DHS**] following campaigns reported to WHO).”

“We included countries based on the following inclusion criteria: i) country was endemic for STH (determined by WHO recommendations for a national deworming program); ii) availability of national and district-level deworming campaign coverage data reported to WHO; and iii) availability of DHS data for corresponding time period (defined by recall period of interviews conducted within the 6 months following campaigns reported to WHO as per DHS question format **used in all countries surveyed**).”

“**As surveys were conducted over an extended period, we** also excluded **individual** DHS observations with **their individual** survey administration date outside the recall period **following** any campaign reported to WHO for their district of residence **as deworming captured by these excluded observations could not refer to a known source of programmatic coverage.**”

“We conducted a series of sensitivity analyses on key parameters and assumptions to determine the robustness of our findings. In one such analysis, we adjusted DHS survey inclusion by varying the recall period assumed for maternally-reported response (base case of 6 months) **across a short-term period of 4 to 8 months that we believed would still yield reliable DHS responses to child deworming receipt to evaluate cases when maternal recall was shorter or longer than designated by the original** **question.**”

*Comment #8*

The sentence “we excluded any deworming campaigns with a reported coverage to WHO over 95%...” is not clear – does this mean you excluded the involved districts from the analysis completely, or that you used the DHS data for those districts but not the WHO data somehow? Please specify in the manuscript.

Response: We appreciate the opportunity to clarify our methods and have revised the sentence with added clarity. At the step outlined, we aimed to determine which deworming campaigns reported to WHO would be included in the final dataset used to estimate coverage reported to WHO. As it was possible for a district to report multiple deworming campaigns with only one campaign excluded due to reliability concerns, we do not exclude the entire district. However, in cases where only one campaign is reported in a district, the district would be excluded from the analysis. The steps to estimate district-level coverage from campaign-level coverage are outlined in the *Statistical Analysis* section of our methods.

-In Methods:

“**This produced a pair of coverage estimates (one DHS estimated and one reported to WHO) only for districts where 1) a campaign was reported to WHO and 2) any DHS responses were collected within the recall period following the campaign reported to WHO.”**

“We excluded **data from any** deworming campaigns with a reported coverage to WHO over 95% from **calculations of district-level coverage reported to WHO** given concerns regarding the data reliability, although we varied this reliability threshold in the sensitivity analysis (27).”

“The primary study outcome was reported deworming receipt by data source; we estimated pairs of deworming coverages and the mean absolute difference in district-level coverage reported to WHO and estimated by DHS **only for districts with both DHS data and data reported to WHO available.**”

*Comment #9*

Please state the source for the population estimates (including age-specific estimates) used as the denominators for the WHO coverage calculations, as well as the sources of the population estimates used in the DHS study designs.

Response: We thank the reviewer for this comment and have updated our manuscript to clarify sources of population data. The population health data used to estimate coverage reported to WHO was also reported to WHO. The denominator for DHS coverage estimates came from the number surveyed.

-In Introduction:

“DHS are large, nationally representative surveys of person-level data on a wide range of health measures from low- and middle-income countries **designed in coordination with governmental organizations, such as a National Statistical Office or Ministry of Health (24).**”

-In Methods:

“WHO publishes **age-stratified, campaign** coverage data for preventive chemotherapy programs for STH, lymphatic filariasis, onchocerciasis, schistosomiasis, and trachoma at the administrative level one (equivalent to district-level) in the Preventive Chemotherapy and Transmission Control databank (PCT), Preventive Chemotherapy Data Portal and Global Health Observatory (17–19,29).”

“WHO data provided deworming coverage by age group (e.g. pre-school aged children) for each campaign, the date of deworming campaign initiation, and the location for each campaign. Coverage reported to WHO was computed as the total number of individuals reported to have received deworming medications over the total population of targeted pre-school aged children per district for a single deworming campaign with adjustments to be aligned with DHS data (30).”

“Third, **to represent the annual district-level deworming coverage reported to WHO,** we aggregated child treatment probabilities at a district-level **divided by the total number of children reported to WHO to have received deworming treatment**.”

*Comment #10*

Please clarify what level of “location” campaigns were conducted on – were they district-level, multidistrict, did they sometimes include only part of a district, etc? the reader needs to have confidence that the areas covered are identical when comparing coverage values across the two data sets.

Response: We appreciate the reviewer’s comment and the opportunity to add clarity to our manuscript. We have updated the methodology section to better characterize the data sources.

-In Methods:

“WHO publishes age-stratified, campaign coverage data for preventive chemotherapy programs for STH, lymphatic filariasis, onchocerciasis, schistosomiasis, and trachoma **at the administrative level one (equivalent to district-level)** in the Preventive Chemotherapy and Transmission Control databank (PCT), Preventive Chemotherapy Data Portal and Global Health Observatory (17–19,29).”

“Based on these criteria, we included data from each **state of** Burundi for 2016-2017, **province of** Myanmar for 2015-2016 and **administrative region of** the Philippines for 2017.”

*Comment #11*

DHSs are not conducted primarily in women. There is a men’s questionnaire, a women’s questionnaire, and a household questionnaire. It appears the deworming question is asked in the women’s questionnaire, which I think may be what you meant. Please correct.

Response: We appreciate the comment. We have updated the methods to clarify that the DHS survey module used to report deworming was only present in the women’s survey.

-In Methods:

“DHS are nationally representative surveys **conducted across countries at the household- and individual-level amongst women and men with women 15-49 years of age as the primary respondents**. We used a DHS question asked to mothers **within the women’s module** on whether each of their children received “drugs for intestinal parasites in the last 6 months”, which served as a maternally-reported metric to estimate pre-school aged child deworming coverage receipt (31).”

*Comment #12*

Data analysis: you discuss using weighting to calculate estimated national-level coverage, but there is no discussion of district-level weighting to account for DHS survey design and actual nonresponse. Please specify whether weighting was used for district-level coverage estimates and whether it was your own or DHS weights. It seems unlikely the DHS participants in each district were perfectly representative of its general population.

Response: We appreciate the reviewer’s comments on this methodological detail. We have updated the methods to specify that the weight variables are provided by the DHS with each dataset and are intended to be applied for national-level statistics, but not district-level. Therefore, we only used weights for national estimates.

-In Methods:

“Finally, we estimated the national-level deworming coverage and standard deviation by calculating the mean probability of deworming receipt using DHS data across all districts. **For national-level coverage estimates, we applied DHS-provided survey weights, due to the multi-stage cluster sampling procedure, which are intended to estimate statistics representative at the national-level from sample data** **(24).**”

*Comment #13*

The term ‘deworming coverage’, as used throughout, is problematic without further qualification. It implies, like ‘vaccine coverage’, that at a given moment in time each individual in the country is either covered or not covered, when in truth people fall along a distribution of how long ago they were last dewormed. Deworming coverage is campaign-specific; it doesn’t make sense to talk about e.g. Burundi’s current national deworming coverage, but about its 2017 deworming campaign’s coverage – and even that only if the entire country did a campaign. More specific consistent terminology is needed. Please consider replacing throughout with something like ‘X year national deworming campaign coverage’, “Y year District Z deworming campaign coverage”.

Response: We appreciate the opportunity to add clarity to the terminology referenced throughout this study. We agree with the reviewer that coverage is campaign- and time-specific and should be interpreted as such. The manuscript has been updated to explicitly provide the intended interpretation for the term “deworming coverage”.

-In Methods:

“We calculated deworming coverage in pre-school aged children at the primary administrative level, referred to as “district” throughout the study, using data reported to WHO and measured by DHS. **As deworming coverage is campaign- and time-specific, we refer to the deworming campaign coverage for a specific year and geographic area as the “deworming coverage” throughout the study (e.g. 2016 Burundi national deworming campaign coverage is referenced as “Burundi national-level deworming coverage”).**”

*Comment #14*

I’m not clear on why an area would possibly have more than one official deworming campaign within 6 months of the same DHS. The highest WHO-recommended deworming frequency is biannual ([https://www.who.int/elena/titles/deworming/en/),](https://www.who.int/elena/titles/deworming/en/) in areas with baseline prevalence over 50%. If you truly found many such situations, this requires some further explanation

Response: We thank the reviewer for this comment to expand on this interesting observation found during our analysis. We have updated the discussion section of the manuscript to discuss potential explanations for why a country may conduct biannual mass deworming without 6 months between each campaign.

-In Discussion:

“**Additionally, WHO recommends biannual treatment in countries with higher STH endemicity by assuming a 6-month separation between campaigns, however in some cases (i.e. select districts of Burundi) campaigns were conducted within a shorter time period of one another. This may be due to alignment with other public health campaigns or anticipated seasonal variation in campaign success. To adjust treatment probabilities for repeat coverage over multiple campaigns,** a “treatment correlation” parameter α was derived through a fitting process that was designed to minimize differences between district-level coverage reported to WHO and estimated by DHS (i.e. the optimistic scenario).”

**Results and Conclusion Comments**

*Comment #15*

School-age deworming programs are commonly done in schools. Please consider discussing the implications of this in light of gender-specific school attendance statistics. If these countries have significant gender gaps in school attendance but no gaps in deworming coverage, what is your interpretation of this? (<https://data.unicef.org/topic/education/primary-education/>)

Response: We appreciate the reviewer’s suggestion for expanding on the gender-stratified analysis and believe incorporation of school attendance data may illuminate additional insights. However, as pre-school aged child deworming is conducted outside school-based platforms given the target demographic population’s age, differences in school attendance by gender may not apply to the analysis. We have revised the manuscript to briefly discuss the typical forms of mass drug administration utilized for pre-school aged children.

-In Introduction:

“Preventive chemotherapy, which involves the periodic, coordinated, large-scale administration of single-dose albendazole or mebendazole (i.e., deworming treatment) to groups at risk, is the primary public health strategy to control STH burden by treating prior infections and decreasing worm burden to prevent future morbidity (4,10). **School-aged child deworming is typically conducted through the existing school-based infrastructure (11,12). Meanwhile, since pre-school aged children cannot access these platforms, pre-school aged deworming is primarily conducted through campaign-style Child Health Days (13,14).**”

-In References:

“11.	World Health Organization. Soil-transmitted helminthiases: number of children treated in 2012 [Internet]. World Health Organization; (Weekly epidemiological record 89). Report No.: 13. Available from: https://www.who.int/wer/2014/wer8913.pdf?ua=1&ua=1

12.	Anderson RM, Truscott JE, Pullan RL, Brooker SJ, Hollingsworth TD. How Effective Is School-Based Deworming for the Community-Wide Control of Soil-Transmitted Helminths? PLoS Negl Trop Dis [Internet]. 2013 Feb 28 [cited 2020 Mar 25];7(2). Available from: https://www.ncbi.nlm.nih.gov/pmc/articles/PMC3585037/

13.	World Health Organization. Schistosomiasis and soiltransmitted helminthiases: number of people treated in 2015 [Internet]. World Health Organization; 2016. (Weekly epidemiological record 91). Report No.: 49. Available from: https://apps.who.int/iris/bitstream/handle/10665/251908/WER9149_50.pdf?sequence=1

14.	Kumapley RS, Kupka R, Dalmiya N. The Role of Child Health Days in the Attainment of Global Deworming Coverage Targets among Preschool-Age Children. PLOS Neglected Tropical Diseases. 2015 Nov 6;9(11):e0004206.”

*Comment #16*

It’s not clear which dataset you’re attributing ‘recall bias’ from albendazole distribution by non-deworming programs to (although I think it’s misclassification bias, not recall bias, no?) Please specify- it seems more intuitive that maternally reported DHS data would be subject to this, not official district deworming records, so I’d expect that to have led to DHS data overestimating coverage, not the other way around. Unless you’re saying that you think mothers are systematically misclassifying deworming campaigns as LF campaigns? Would state this the first time you discuss this source of bias (in Methods) if so.

Response: We thank the reviewer for their comment and have updated the methods to clarify the aim to use lymphatic filariasis data as a proxy to study misclassification of deworming treatment for any non-STH related program. Indeed, recall bias only applies to the DHS self-reported data. We further revised use of the phrase “recall bias” in response to comment #18.

-In Methods:

“**Misclassification of deworming treatment for only lymphatic filariasis** served as a proxy for misclassification due to distribution of albendazole for **any** non-deworming public health programs.”

“We performed an analysis of coverage differences across data sources, after exclusion of children below 2 years of age, stratified by district-level treatment of lymphatic filariasis **using albendazole and DEC (applicable to children 2-5 years)** to serve as a proxy to assess misclassification due to non-deworming public health programs distributing albendazole (see Appendix)

- In Discussion

“The study also highlighted key challenges to integrating DHS and WHO data for deworming coverage estimates including reliability of systems reporting to WHO, accuracy of DHS self-reported deworming receipt, and concurrent medication by public health programs using albendazole, **such as for lymphatic filariasis** (where mothers may **misclassify** treatment **as only preventive** for lymphatic filariasis **and not as a deworming treatment**).”

“The difference in data compatibility between WHO and DHS data for Burundi, Myanmar and the Philippines may be partially explained by variation in the quality of national data systems reporting to WHO and recall **inaccuracies in the DHS survey due to self-reporting or due to misclassification of** albendazole receipt **during other public health** programs (e.g. albendazole for lymphatic filariasis) or **from** non-programmatic distribution (e.g. albendazole given in clinic).”

*Comment #17*

Please fix ‘Center for Disease Control’ – it is ‘U.S. Centers for Disease Control and Prevention’.

Response: We thank the reviewer for noticing this error and have updated the agency name in the manuscript.

-In Discussion:

“We established the data reliability threshold value of 95% using a previous analysis of validation surveys by the **U.S. Centers for Disease Control and Prevention** for deworming campaigns from 2000-2011, where 0 of 16 surveys conducted had estimated coverage values above 95% despite reports to the WHO for 7 of the 16 campaigns (27).”

“Deworming coverage validation surveys have previously been conducted by the **U.S. Centers for Disease Control and Prevention** and Hellen Keller International immediately following national deworming campaigns (27,28).”

*Comment #18*

The discussion of maternal recall and recall bias seems problematic – the gestalt one gets is that mothers are being blamed for giving the ‘wrong’ answer, but their answers appear in the examples given to either not be wrong at all (correct knowledge that their own child got unprogrammed/unintended deworming) or be misclassification bias rather than recall bias. I suspect an individual mother’s recall of her child’s deworming status is likely to be much more accurate than official aggregate figures. Certainly in the vaccination world, surveys of individual immunization documentation yield far lower coverages than values reported to WHO (“administrative coverage”) and I think are considered more accurate.

Response: We thank the reviewer for this important comment, and agree we should not assign blame to mothers when the correct coverage estimate is not clear. We believe that the most likely sources of recall bias (mis-association of albendazole with only LF or consideration of non-programmatic receipt) may yield a misclassification of the child’s deworming status as the reviewer mentioned. We have updated the manuscript to clarify at all mentions of recall bias that this may cause a misclassification of deworming status, and we have additionally adjusted the language surrounding maternal reporting to reduce the potential for blame. Additionally, we have expanded the discussion section to discuss alternative explanations for the variation in coverage estimates using DHS data and reported to WHO.

-In Discussion:

“The study also highlighted key challenges to integrating DHS and WHO data for deworming coverage estimates including reliability of systems reporting to WHO, accuracy of DHS self-reported deworming receipt, and concurrent medication by public health programs using albendazole, such as for lymphatic filariasis (where mothers may **misclassify** treatment **as only preventive** for lymphatic filariasis **and not as a deworming treatment**).”

“The difference in data compatibility between WHO and DHS data for Burundi, Myanmar and the Philippines may be partially explained by variation in the quality of national data systems reporting to WHO and **recall inaccuracies in the DHS survey due to self-reporting or due to misclassification of** albendazole receipt **during other public health** programs (e.g. albendazole for lymphatic filariasis) or **from** non-programmatic distribution (e.g. albendazole given in clinic).”

“In the case of Myanmar, regional endemicity for lymphatic filariasis **potentially** increased misclassification present within coverage estimates by DHS data as **maternally-reported** **deworming receipt was much lower than** reports to WHO of albendazole receipt, possibly **from only associating** the treatment **with** its indication of lymphatic filariasis (applicable for children 2-5 years) and not for deworming. While there may be biases present in DHS data from including unprogrammed sources of deworming receipt, not otherwise present in data reported to WHO, this would bias estimates by the DHS to have over-estimated deworming coverage which would not explain the observed pattern of discrepancies. **On the other hand, in the case of Burundi, exclusion of 30% of campaigns reported to WHO due to data reliability concerns (coverage >95%) may indicate an under-reporting of the target population size (e.g. use of outdated census data) to WHO or over-reporting of doses delivered (e.g. doses delivered outside target age range) to WHO.**”

“As DHS data relied on maternally-reported child deworming receipt, it is **potentially** subject to **misclassification of treatment arising from recall bias**. However, recent work suggests maternally-reported child deworming data are a reliable estimate of programmatic coverage in the short-term period following medication, with reported estimates within 5-10% of official campaign estimates **and with vaccine coverage estimates by WHO routinely incorporating maternally-reported survey data (39,40).**”

“**The proposed secondary source of deworming data, DHS,** relied on maternally-reported child deworming receipt, which **are potentially** subject to recall bias as discussed above.”

“As lymphatic filariasis coverage data are often reported for all age groups and are not specific to pre-school children, **misclassification of** albendazole receipt for **only** lymphatic filariasis may have a smaller effect than found given we assumed all districts receiving treatment will include pre-school aged children in coverage.”

*Comment #19*

From what I can tell, MDAs for LF do not routinely include all pre-school-aged children (<https://www.cdc.gov/mmwr/preview/mmwrhtml/mm6223a3.htm> – children under 2 years excluded). Please consider what effect this has on your calculations and interpretations.

Response: The reviewer is correct that LF campaigns do not include children <2 years. In performing the sensitivity analysis for coverage differences stratified by LF endemicity, we ensured to only include deworming data for pre-school aged children over 2 years of age (data of children 2-4 years included). We updated the manuscript methods to clarify this point and added language in the discussion section at first mention of potential misreporting due to LF treatment to clarify the ages included.

-In Methods:

“We performed an analysis of coverage differences across data sources**, after exclusion of children below 2 years** of age**,** stratified by district-level treatment of lymphatic filariasis **using albendazole and DEC (applicable to children 2-5 years)** to serve as a proxy to assess misclassification due to non-deworming public health programs distributing albendazole (see Appendix)”

-In Discussion:

“In the case of Myanmar, regional endemicity for lymphatic filariasis potentially increased misclassification present within coverage estimates by DHS data as maternally-reported deworming receipt was much lower than reports to WHO of albendazole receipt, possibly from only associating the treatment with its indication of lymphatic filariasis **(applicable for children 2-5 years)** and not for deworming.”

*Comment #20*

Not clear what your statement about ‘compatibility limitations’ in Myanmar means – are you just saying the DHS coverage estimates there had wide confidence intervals due to the small sample size and the WHO estimates fell within those, so you don’t know if the discrepancies are real? Would clarify.

Response: We appreciate the opportunity to clarify that our intended meaning of ‘compatibility limitations’ of the data sources in Myanmar was that the dates of the DHS survey and WHO reported campaigns did not line up well enough to perform a sufficient comparison.

-In Discussion:

“Myanmar presents a case study for compatibility limitations **due to a much smaller DHS sample size (<150 samples per district)** those used **for** Burundi or the Philippines **which may limit the sub-national representation of deworming estimates from DHS data and bias the wide discrepancies in coverage estimates by data source.** This was due to a smaller DHS campaign, fewer children per mother interviewed, and a very limited alignment of the DHS sampling period with the recall period for deworming campaigns **reported to WHO.**”

*Comment #21*

Please expand the final conclusion if possible. The reader has learned that in some countries, DHS and WHO coverage values were quite close, and in others they weren’t. Authors state that that means DHS data should perhaps be used going forward, but how exactly? Is your idea that if discrepancies are over a certain, a more thorough quality review of the national reporting system is needed?

Response: We thank the reviewer for their advice to describe potential applications of DHS deworming data with added clarity. We have updated our concluding paragraph to describe two potential opportunities for incorporation of the DHS deworming data.

-In Discussion:

“In this study, we found that district-level deworming coverage estimates for pre-school aged children reported to WHO and estimated by DHS were broadly consistent across Burundi, highly discrepant in Myanmar, and varied in alignment across the Philippines. As deworming coverage remains a key metric in monitoring and evaluating programs against STH globally, our study indicates that incorporation of DHS for deworming coverage **for quality control**, under specific settings, may improve **data quality and resource allocation by systematically classifying the urgency of coverage validation surveys for each national reporting system. Additionally, where coverage estimates are compatible, DHS deworming data can support the development of novel deworming coverage equity metrics, such as by geography and gender.**”

**Response to Reviewer #2**

**General Comments**

*Comment #1*

This paper is a highly relevant one and calls attention to the limitation of reported MDA coverage to WHO as basis for indicating progress of morbidity control in STH infections. The methods are found to be generally rigorous, and the results provide an excellent view of DHS data to complement the use of reported MDA data to WHO to help track progress.

Response: We thank Reviewer #2 for their kind words and helpful responses.

**Methods Comments**

*Comment #2*

Methodology was found in order.

Response: We appreciate Reviewer #2’s time to evaluate our methodology section.

**Results Comments**

*Comment #3*

Results are clearly presented.

Response: We appreciate Reviewer #2’s time to evaluate our results section.

**Conclusion Comments**

*Comment #4*

Conclusions are appropriate, and limitations are highlighted. May I suggest a mention of variability in reporting resulting from a decentralized health system, where districts are largely left on their own to implement mass deworming strategy. Variable MDA coverage may well be used as part of advocacy in enhancing service delivery, outputs and outcomes of peripheral health units.

Response: We thank the reviewer for this insightful comment and agree that many of the countries endemic to STH likely utilize a decentralized health system for deworming coverage. We have updated the manuscript discussion to discuss this potential cause of sub-national variation.

-In Discussion:

“This analysis highlighted opportunities to identify sub-national regions with low coverage that may merit further investigation. **Variations in sub-national coverage per country may be attributed to the use of decentralized health systems where sub-national regions are left to apply their own methods for implementing deworming activities.** Overall, the findings support the utilization of sub-national DHS deworming data under specific settings to complement data reported to WHO by serving as an independent source of geographically and demographically disaggregated coverage data for pre-school age children.”

**Editorial and Data Presentation Comments**

*Comment #5*

Minor revision in the discussion is recommended to include the comment made above.

Response: We appreciate the comments and have revised our discussion section accordingly as noted in response to Comment #4.
