# Supplementary material for: Comparison of World Health Organization and Demographic and Health Surveys data to estimate sub-national deworming coverage in pre-school aged children
Source: PLoS Negl Trop Dis. 2020 Aug 17;14(8):e0008551. doi: 10.1371/journal.pntd.0008551 (PMC7462292; doi:10.1371/journal.pntd.0008551)
Supplement: Attachment — Submitted filename: Lo- PNTD Response to Editor R2.docx [file pntd.0008551.s015.docx]

“Comparison of World Health Organization and Demographic and Health Surveys data to estimate sub-national deworming coverage in pre-school aged children” (reference no. PNTD-D-20-00061) – Point-by-point response

21 May 2020

Dear Editor,

We are pleased to submit a revised version of our Research Article, entitled “Comparison of World Health Organization and Demographic and Health Surveys data to estimate sub-national deworming coverage in pre-school aged children” (reference no. PNTD-D-20-00061). We thank you and the two external reviewers very much for the careful examination and helpful comments, and for the invitation to submit a re-revised manuscript. We have addressed the issues brought forward, with a concerted effort on adding clarity to the terminology used and to our methodologies. Below, please find our point-by-point response, indicating the changes made in the manuscript.

We believe this article is timely in providing a method to validate coverage progress in time for the approaching 2020 WHO Roadmap deadline and ahead of transitioning to the 2030 WHO Roadmap. We trust that our findings will be of interest to the readership of PLOS Neglected Tropical Diseases, and will support efforts to improve data accuracy and to develop novel metrics to assess geographic variation and equity in coverage globally.

Sincerely,

Nathan C. Lo, MD PhD

University of California, San Francisco

On behalf of co-authors

**Response to Reviewer #1**

**General Comments**

*Comment #1*

General: Please read over for grammar, syntax and precise meaning. E.g. constructions like in the abstract, “the progress of deworming programs to control” (should be “in controlling”). Or in the abstract conclusion section, “deworming coverage…was heterogeneous across countries”. I think you mean something more like “Matching between deworming coverages reported to WHO…was heterogeneous…” Or in the introduction, “There is growing interest to utilize” – should be “in utilizing”. Or in the Discussion: “our analysis found coverage differences…may be compatible”. (The differences aren’t compatible; perhaps the estimates are compatible or comparable.) Etc.

Response: We appreciate the reviewer’s attention to improving the writing of the manuscript. We have revised throughout with a few examples illustrated below.

-In Abstract:

“The key metric for monitoring the progress of deworming programs **in controlling** soil-transmitted helminthiasis (STH) is national drug coverage reported to the World Health Organization (WHO).”

“**Agreement of deworming** coverage reported to WHO and estimated by DHS data was heterogeneous across countries, varying from broadly **compatible** in Burundi to largely discrepant in Myanmar.”

-In Introduction:

“There is a growing interest **in utilizing** sub-national data to develop programmatic metrics to better understand geographic variation, gender parity, and other inequities.”

-In Discussion:

“Yet, comparatively, our analysis found coverage **reported to WHO and estimated by DHS data may be** compatible in some settings.”

*Comment #2*

Introduction (and wherever else this term appears): “prior” infections – perhaps “existing” instead? Deworming is not treating infections that happened in the past and have resolved, but rather current ongoing infections.

Response: We appreciate the reviewer’s attention to detail and have updated the manuscript’s language surrounding the role of preventive chemotherapy.

-In Introduction:

“Preventive chemotherapy, which involves the periodic, coordinated, large-scale administration of single-dose albendazole or mebendazole (i.e., deworming treatment) to groups at risk, is the primary public health strategy to control STH burden by both treating **existing** infections and decreasing worm burden to prevent future morbidity (4,10).”

*Comment #3*

Introduction: “the key metric…remains national treatment coverage” – perhaps ‘national campaign coverage”, since the prior paragraph was all about the campaigns and you’re referring to them here? Otherwise could be misunderstood as newly introducing the idea of clinical treatment based on symptoms.

Response: We appreciate the comment and have updated the language in the introduction to clarify the metric used to monitor programmatic deworming and to bring it in line with the terms used earlier in the manuscript.

-In Introduction:

“The key metric for monitoring the progress of deworming programs remains national **drug** coverage (%) in school-age and pre-school aged children”

**Methods Comments**

*Comment #4*

Overview: Despite the definition now provided for “recall period”, it remains confusing throughout, because the definition doesn’t distinguish between “how far back we asked the respondent to think back in answering the question” and “how far back the respondent actually thought in answering the question”, which is the critical issue. To make things harder, I believe the actual most common understanding of that phrase is neither, but is more like “how far back the respondent can reliably remember things”. Please revise the definition to clarify that (if I’m correct) you mean the first option. Perhaps consider whether you need to create distinct terms for this and the other plausible meaning(s) you use later in the ‘sensitivity analysis’ section. E.g. “requested recall period” vs “actual recall period” etc.

Response: We appreciate the reviewer’s comment and have restructured the *Overview* to provide additional details on why the recall period was constructed and its intended interpretation. We have also reviewed the remainder of the manuscript to ensure descriptive terms (e.g. “true” or “assumed”) followed recall period where appropriate.

-In Methods:

“The coverage data were matched geospatially (at district-level) and temporally (aligning DHS administration dates with the recall periods following campaigns reported to WHO). **Recall period, the time period during which reports of treatment in DHS are likely to align with a specific deworming campaign, was defined as 6 months, since in the DHS, mothers were asked if their child received deworming during the previous 6 months.”**

“We adjusted DHS survey inclusion by varying the recall period assumed for maternally-reported response (base case: 6 months) across a short-term period of 4 to 8 months that we believed would still yield reliable DHS responses to child deworming receipt to evaluate scenarios when **the true** maternal recall might have been shorter or longer than designated by the actual survey question.”

*Comment #5*

Data source: While inclusion criteria are clearer, we’re not all the way there yet. The “Data Source” paragraph does not answer: 1) Were countries included if they had matching DHS and WHO data for only part of the country? 2) Could a given district be included more than once if it had matched pairs of DHS and WHO data for two different years? (e.g. for one DHS and also its next DHS).

Response: We appreciate the reviewer’s comments and have included language in the *Overview*, *Data Source*, and *Statistical Analysis* sections to clarify that the analysis can be conducted so long as data from DHS and WHO are available for a subset of districts. No district can be included twice as DHS samples are collected on average five-year interval.

-In Methods:

“This produced a pair of coverage estimates (one DHS estimated and one reported to WHO) only for **the subset of districts in each country** where 1) a campaign was reported to WHO and 2) any DHS responses were collected within the recall period following the campaign reported to WHO.”

“We reviewed a complete list of countries with available district-level deworming coverage data available from WHO (using PCT and GHO databanks) and DHS (using Phase VII surveys). We included countries based on the following inclusion criteria: i) country was endemic for STH (determined by WHO recommendations for a national deworming program); ii) availability of national and district-level deworming campaign coverage data reported to WHO; and iii) availability of individual-level DHS data for the corresponding time period (defined by recall period of interviews conducted within the 6 months following campaigns reported to WHO as per DHS question format used in all countries surveyed) **for at least a subset of districts reporting to WHO**.”

“We estimated pairs of deworming coverages and the mean absolute difference in district-level coverage reported to WHO and estimated by DHS for **the subset of districts per country** with both DHS data and data reported to WHO.”

*Comment #6*

Optionally, you could also consider additionally stating (since it appears this is what happened with the Philippines) something like: “Data on different subnational regions from a single national DHS could potentially be matched with WHO data from one national campaign in one region but the next national campaign (six months later) in another region, if the DHS data collection took several months to progress from the first region to the second.”

Response: We appreciate this comment and have updated the methodology and figure descriptions to better reflect this possibility while also balancing attempts to streamline the methodology. As a clarification, while two national deworming campaigns did overlap in the Philippines when we use an 8-month recall period for DHS data (at which point we used the coverage optimization algorithm), in the base case (6 month recall period) all DHS surveys aligned with only one national deworming campaign in the Philippines.

-In Methods:

“For districts where only one deworming campaign reported to WHO **overlapped with the recall period following DHS sampling**, we used the corresponding campaign coverage as the treatment probability for each child targeted for treatment. For districts where **the recall period for** two deworming campaigns were reported to WHO **overlapped with** **DHS** **administration dates**, we used a “treatment correlation” parameter, α, to combine coverage reported to WHO from all applicable campaigns into a single treatment probability for all children targeted for treatment (See Appendix).”

-In Figures:

“Figure 1. Timeline of **national** deworming campaigns reported to WHO for pre-school aged children and DHS data collection periods in study countries. We obtained sub-national deworming coverage data reported to WHO and estimated by DHS across comparable timelines. We used WHO data on programmatic deworming to determine **national** deworming campaign start dates (dates on left-hand side and time-axis in month/year, e.g. 06/2016 is June 2016) and estimated coverage for Burundi (panel A), Myanmar (panel B) and the Philippines (panel C). We used DHS data from each country to estimate deworming coverage by matching respondents to the corresponding deworming campaigns, based on the survey date and recall period in the orange/gray bars (base, 6-month recall; alternatively, 4- or 8-months based on the sensitivity analysis) that represents the accepted time period (months) after deworming campaign start. **Note: Overlap of multiple deworming campaigns at the sub-national level may vary from the overlap presented at the national level.**”

*Comment #7*

Also optionally, for maximal clarity, you might also consider reorganizing this paragraph to state first the country-level inclusion criteria already provided, then the district-level inclusion criteria, then the individual-level criteria (currently provided later in the Methods section).

Response: We appreciate this comment and have updated the *Data Source* section to further clarify this intended flow. We specify that the district-level inclusion criteria are only applicable for data reported to WHO as the databank records are aggregated to this unit. Additionally, we clarify individual-level inclusion criteria are only applicable for DHS data as the sampling is conducted at an individual-level.

-In Methods:

“We included countries based on the following inclusion criteria: i) country was endemic for STH (determined by WHO recommendations for a national deworming program); ii) availability of national and district-level deworming campaign coverage data reported to WHO; and iii) availability of **individual-level** DHS data for the corresponding time period (defined by recall period of interviews conducted within the 6 months following campaigns reported to WHO as per DHS question format used in all countries surveyed) for at least a subset of districts reporting to WHO.”

“WHO publishes age-stratified, campaign coverage data **at the administrative level one (equivalent to district-level)** for preventive chemotherapy programs against STH, lymphatic filariasis, onchocerciasis, schistosomiasis, and trachoma in the Preventive Chemotherapy and Transmission Control databank, Preventive Chemotherapy Data Portal and Global Health Observatory (17–19,29).”

*Comment #8*

Data source: Optionally, it would be of interest to tell us how many countries have deworming coverage data available from WHO, and how many from DHS. This would better contextualize the ultimate inclusion of only three.

Response: We thank the reviewer for this suggestion and agree inclusion of this data may support the discussion on the limited overlap between DHS and WHO datasets. However, because the number of countries engaging in programmatic deworming varies over time (e.g. Country may report data in 2015 and not in 2016), it is difficult to define a time period in which we can succinctly summarize the number of countries with available data per data source. In place, we have updated the methodology to further specify the data sources used (e.g. which DHS survey phase to use) to allow readers to compare overlap of datasets for themselves as needed.

-In Methods:

“We reviewed a complete list of countries with available district-level deworming coverage data available from WHO **(using PCT and GHO databanks)** and DHS **(using Phase VII surveys)**.”

**Conclusions Comments**

*Comment #9*

“The difference in data compatibility” – confusing; I think you mean “different in coverage estimate values” or something.

Response: We appreciate the reviewer’s comment and have updated the language in the discussion to clarify when we were comparing compatibility of datasets and when we were comparing compatibility of coverage estimates.

-In Discussion:

“The difference in **agreement of coverage estimated using data reported to WHO and from DHS for** Burundi, Myanmar and the Philippines may be partially explained by variation in the quality of national data systems reporting to WHO and recall inaccuracies in the DHS survey due to self-reporting or due to misclassification of albendazole receipt from other public health programs”

“The broad compatibility of **coverage estimated by DHS data with reports** to WHO in Burundi was particularly notable”

“we assumed 67.8% of all children covered were treated by both deworming campaigns—an assumption that is unlikely to have occurred as the parameter was fit for the most optimistic case of **coverage estimate compatibility**”

*Comment #10*

Please briefly discuss the size and direction of bias potentially introduced by the individual-level DHS observation inclusion criteria – what were the response rates for these three DHSs? Did the DHS report authors record any concerns about their representativeness? Are households who were found by and agreed to participate in DHS likely/known to be different in key demographic ways from those not found/refusing? Would those e.g. have made them more likely to have also been “found” by deworming campaigns, maybe biasing both sets of estimates upward, or not?

Response: We appreciate the reviewer’s comments and have expanded the discussion section to discuss the potential impact of non-response bias and how it was handled in the analysis.

-In Discussion

“As DHS data relied on maternally-reported child deworming receipt, they are potentially subject to misclassification of treatment arising from recall bias. However, recent work suggests maternally-reported child deworming data are a reliable estimate of programmatic coverage in the short-term with reported estimates within 5-10% of official campaign estimates, and with vaccine coverage estimates by WHO routinely incorporating maternally-reported survey data (39,40). **Additionally, as DHS coverage is estimated using survey sample data, non-response biases may affect the sample representativeness, which is not the case with data reported to WHO. However, as the response rates for all three countries were over 90% (with no indications of differential non-response) and survey weights were applied for national-level comparisons, we believe DHS data are likely representative at least at the national-level (41–43)**.”

-In References:

“41.	Philippine Statistics Authority - PSA, ICF. Philippines National Demographic and Health Survey 2017 [Internet]. Quezon City, Philippines: PSA and ICF; 2018. Available from: http://dhsprogram.com/pubs/pdf/FR347/FR347.pdf

42.	Ministry of the Presidency in charge of Good Governance and Planning - MPBGP, Ministry of Public Health and the Fight against AIDS - MSPLS, Institute of Statistics and Economic Studies of Burundi - ISTEEBU, ICF. Burundi Third Demographic and Health Survey 2016-2017 [Internet]. Bujumbura, Burundi: MPBGP, MSPLS, ISTEEBU, and ICF; 2017. Available from: http://dhsprogram.com/pubs/pdf/FR335/FR335.pdf

43.	Ministry of Health and Sports - MoHS, ICF. Myanmar Demographic and Health Survey 2015-16 [Internet]. Nay Pyi Taw, Myanmar: MoHS and ICF; 2017. Available from: http://dhsprogram.com/pubs/pdf/FR324/FR324.pdf”

*Comment #11*

In the limitations, please briefly address the inclusion of only three countries. This seems inevitable, given you need a close time match and DHS happen only every 5 years, and you were somewhat lucky to get countries in three different regions, but are these three likely to be representative of the many other countries where deworming happens? Optionally, does this difficulty finding time matches suggest that perhaps STH campaigns could be deliberately timed to closely precede an upcoming DHSs in countries where MoHs want to use DHS data for coverage validation?

Response: We have updated the discussion section to briefly discuss our recommendation that the analysis should be interpreted as a set of scenarios for data compatibility as opposed to a globally representative analysis. This is further expanded upon in the concluding paragraph.

-In Discussion:

“The study results should be taken in context with the limitations of the data and assumptions of the analysis (see Appendix). **As only three countries were included, due to current challenges aligning DHS and WHO datasets available, the analysis presents unique scenarios of compatibility but is unlikely to be globally representative of the compatibility of data reported to WHO and by DHS.”**

*Comment #12*

Biannual – I think you mean semiannual (twice per year, not once every two years).

Response: We appreciate the reviewer’s suggestion and have updated the language used in the discussion section accordingly.

-In Discussion:

“Additionally, WHO recommends **semiannual** treatment in countries with higher STH endemicity, assuming a 6-month separation between campaigns.”

“Furthermore, sensitivity analysis found that this poorly characterized parameter will likely affect the study findings in settings with **semiannual** treatment”

**Response to Reviewer #2**

**General Comments**

*Comment #1*

Generally an acceptable paper for dissemination.

Response: We thank reviewer #2 for their kind support of our work.

**Methods Comments**

*Comment #2*

Objectives and methods are clearly described. Statistical methods and analysis are well noted.

Response: We thank reviewer #2 for their efforts to once more review the study methodology.

**Results Comments**

*Comment #3*

Results are clearly laid out and of sufficient clarity.

Response: We thank reviewer #2 for their efforts to once more review the study results.

**Conclusion Comments**

*Comment #4*

Conclusions are well supported by data presented, and public health relevance of the study is included.

Response: We thank reviewer #2 for their efforts to review the study discussion and their kind words in support of the conclusions drawn.
